# Photo-Rechargeable Sodium-Ion Batteries with a Two-Dimensional MoSe_2_ Crystal Cathode

**DOI:** 10.1021/acs.nanolett.4c03471

**Published:** 2025-01-15

**Authors:** Gang Cheng, Zhenyu Guo, Nagaraju Goli, Filip Podjaski, Kaitian Zheng, Jinglin Jiang, Sami Ramadan, Gwilherm Kerherve, Stefano Tagliaferri, Mauro Och, Norbert Klein, Mattia Cattelan, Stefano Agnoli, Maria-Magdalena Titirici, Cecilia Mattevi

**Affiliations:** † Department of Materials, Imperial College London, London SW7 2AZ, United Kingdom; ‡ Department of Chemical Engineering, Imperial College London, London SW7 2AZ, United Kingdom; § Department of Chemistry and Centre for Processable Electronics, Imperial College London, 80 Wood Lane, London W12 7TA, United Kingdom; ∥ Department of Chemistry and INSTM Padua Research Unit, University of Padua, Via Marzolo 1, 35131 Padova, Italy

**Keywords:** MoSe_2_, exfoliation, Na-ion battery, capacity, photocharging

## Abstract

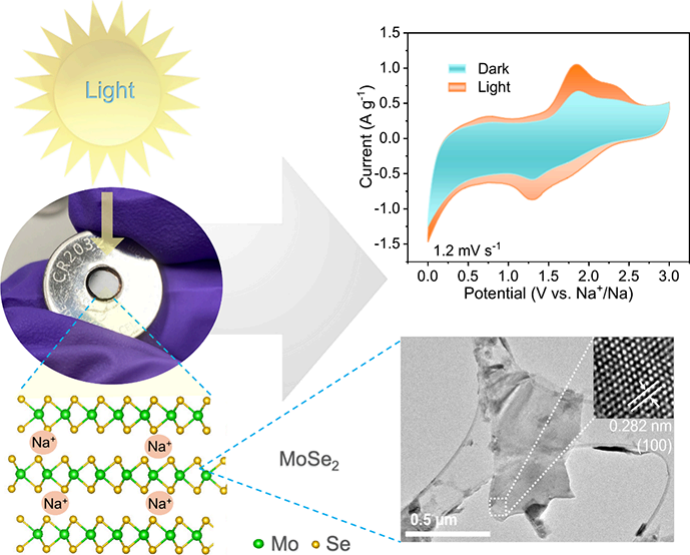

Combining energy harvesting with energy storage systems in a single device could offer great advantages for continuous power supply in both indoor and outdoor electric applications. In this work, we demonstrate a photochargeable sodium-ion battery (PSIB) based on a photoactive cathode of two-dimensional crystals of MoSe_2_. This photocathode enables spontaneous photodriven charging of a sodium-ion battery cathode under illumination and an increase in the reversible capacity to 29% at 600 mA g^–1^ compared to that under dark conditions during galvanostatic cycling. Exposure of MoSe_2_ to light drives the Na^+^ extraction, prompted by photogenerated holes, and accelerates the charge transfer kinetics with improved ion diffusion, which leads to an increased capacity. Moreover, the PSIB can be charged to 1.68 V under light illumination without applying an external current. Our work paves the way for the development of light-driven rechargeable batteries, which can benefit off-grid technologies such as the Internet of Things.

Solar energy conversion is envisioned to play a key role in reducing greenhouse gas emissions, cutting exposure to air pollutants and improving energy security.^1−3^ As batteries are now coupled with solar cells to compensate for intermittency, there is an increased demand for reducing the volume, weight, and cost of such devices.^4,5^ The need to power batteries in remote areas off-grid, the increasing demand of structural batteries for the automotive industry, and the development of the Internet of Things (IoT) sensor networks,^6−8^ which rely on small portable batteries to operate, are increasing the demand to find new solutions to couple solar energy conversion with energy storage. For instance, in the case of IoT platforms, charging and replacing batteries usually lead to an increased level of power consumption and the disturbance of data transfer.^9,10^ Thus, integrating a solar cell with a rechargeable battery into one device design in which either the anode or the cathode of the battery acts as a photo-absorber enabling charge separation inside the material would be transformative.^11,12^ Moreover, there is increased interest in harvesting energy from not only solar light but also artificial light sources,^13^ such as artificial illumination in the form of light-emitting diodes (LEDs) and ambient diffuse light.^13,14^ Sodium-ion batteries (SIB) make up a promising class of batteries due to the earth abundance of sodium, which makes them an appealing alternative to lithium-ion batteries.^15−21^ Currently, efforts to demonstrate batteries that can be charged directly by light without the need for external power sources or solar cells have been limited to zinc-ion batteries and lithium-ion batteries.^18,22−24^

Here we report a photo-rechargeable sodium-ion battery using two-dimensional MoSe_2_ nanosheets as a photocathode material enabling its recharging by Na deintercalation. Upon LED illumination, MoSe_2_ photochargeable sodium-ion battery (PSIB) shows exciton separation, which enables charging of the battery half-cell without the application of an external voltage or current. It appears that the photocathode is charged via hole-assisted deintercalation of Na, enabling further reuse for discharge with excess ions from the Na anode. Moreover, we show that the capacity of the MoSe_2_ SIBs is improved by LED light exposure during galvanostatic cycling, with an increase of ∼6% at 60 mA g^–1^ and ∼29% at a specific current of 600 mA g^–1^. The increase in capacity at a high specific current is an indication that light has a more prominent effect in increasing the number of charges generated at the surface of the materials because at high specific currents the capacity mechanism is dominated by surface kinetics phenomena. Controlled experiments allow us to exclude heating effects as being responsible for the enhanced kinetics of the ion transport under illumination. This study can pave the way toward establishing PSIBs as conventional power sources that can be powered by artificial light.

MoSe_2_ is a layered compound that belongs to the family of transition metal dichalcogenides (TMDs)^25,26^ formed by triatomic layers of Mo atoms sandwiched between two Se atoms that are held together by van der Waals forces.^27^ These establish an interlayer spacing of 0.65 nm between two adjacent layers that has been proven to be suitable for Na insertion.^28−30^ Additionally, layered MoSe_2_ is a semiconducting material with an indirect band gap of 1.41 eV,^31^ while in monolayer form, experimental work has demonstrated a band gap of ∼1.58 eV^31^ and presents a theoretical specific capacity of 422.28 mAh g^–1^.^32−35^ Bare MoSe_2_ has been reported to have limited cyclic stability, linked to the decomposition and formation of soluble polyselenides upon Na-ion insertion.^36,37^ However, it has been reported that the cyclic stability can be significantly improved by combining MoSe_2_ with carbon nanospheres, leading to retention of 87% of the capacity after 1000 cycles compared to that with pristine MoSe_2_ (22%).^38^ This combination effectively restrains the dissolution of intermediate polyselenides and mitigates the volume expansion occurring during sodiation and desodiation.^39,40^ A unique physical property of group VI TMDs, including MoSe_2_, is the strong electron–photon interaction that leads to the absorption of ≲20% of the incident light in monolayers in the range between 1.5 and 3 eV,^41,42^ making it appealing for light-harvesting applications.^43^ Two-dimensional MoSe_2_ flakes were produced by solution phase exfoliation using bulk MoSe_2_ powder under ultrasonication in a mixture of isopropyl alcohol (IPA) and deionized water, as shown in Figure 1a (exfoliation process included in the Supporting Information).^44^ The as-prepared MoSe_2_ flakes were then drop-casted from a solution on carbon paper to fabricate the photocathode for the MoSe_2_ SIB (Figure 1b and Figures S1 and S2). The exfoliated MoSe_2_ flakes are homogeneously distributed on the surface of the carbon paper. The crystallinity of the MoSe_2_ flakes is preserved during the exfoliation process as transmission electron microscope (TEM) imaging shows an interplanar (100) spacing of 0.282 nm, and X-ray diffraction (XRD) characterization further evidences the presence of a 2H phase and a homogeneous distribution of Mo and Se via energy-dispersive X-ray spectroscopy (EDS) mapping (Figure 1c,d,f).^45,46^ The 2H nature of MoSe_2_ was further confirmed by X-ray powder diffraction (Figure 1f) and Raman spectroscopy (Figure 1g). The characteristic Raman mode of the out-of-plane (A_1g_) vibration at ∼242 cm^–1^ is identified as well as weaker peaks ascribed to the E_2g_^1^ in-plane vibrational mode between ∼284 and 430 cm^–1^ and overtones (between ∼400 and ∼580 cm^–1^) (Figure 1g).^47^ Finally, using ultraviolet–visible (UV–vis) light absorption, the A and B excitonic transitions characteristic of MoSe_2_ were observed at 756 and 653 nm, respectively (Figure 1h).

**Figure 1:**
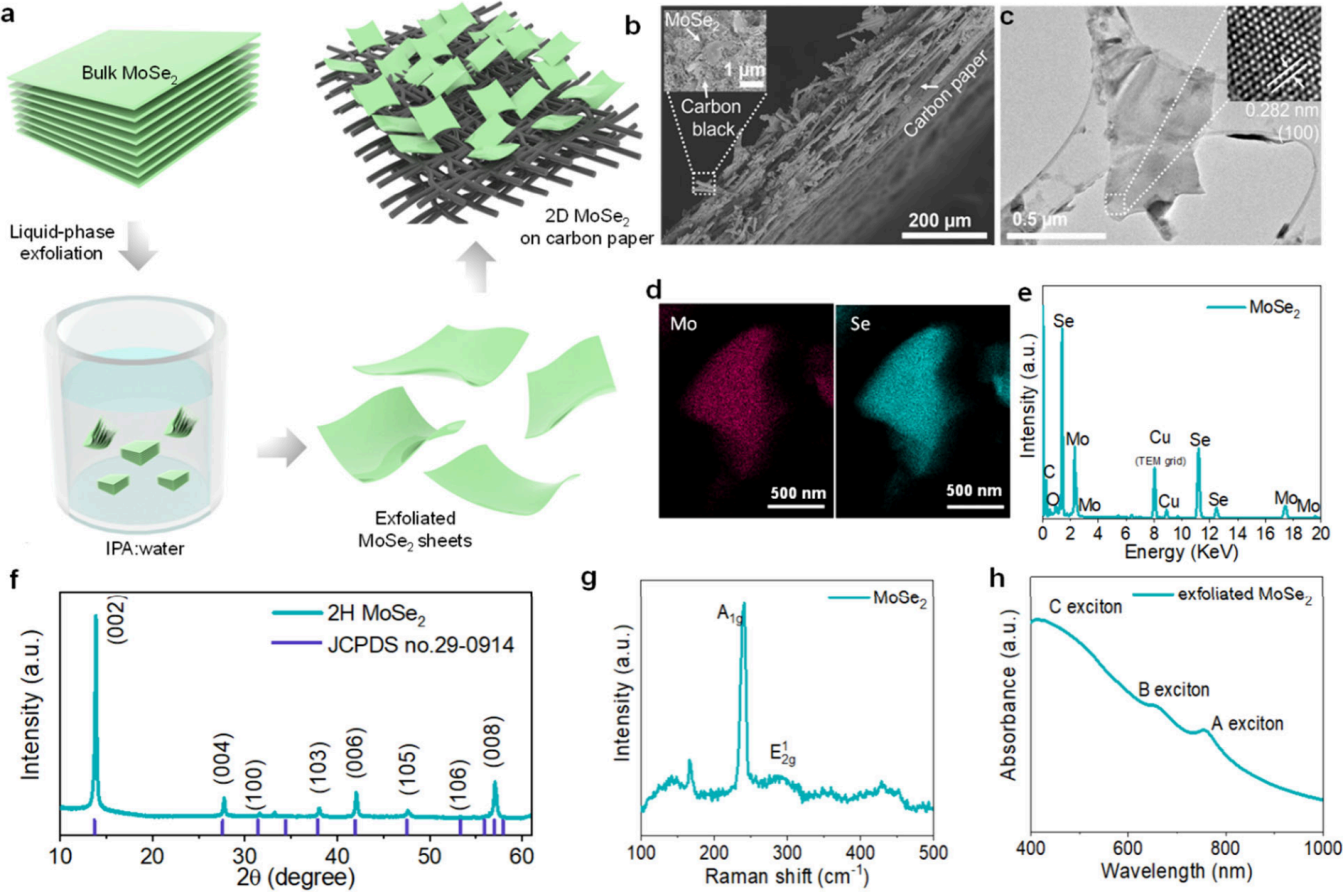
(a) Schematic diagram of the fabrication process for the MoSe_2_ photocathode, from the exfoliation of the bulk powder of MoSe_2_ in IPA:water to the deposition on carbon paper. (b) SEM images of a MoSe_2_ photocathode cast on carbon paper (the inset SEM image shows the randomly distributed MoSe_2_ and carbon black on carbon paper). (c) TEM image of an exfoliated MoSe_2_ flake. (d–h) EDS mapping, EDS elemental spectrum, XRD pattern, Raman spectrum, and UV–vis spectrum, respectively, of exfoliated MoSe_2_ in the form of thin films.

First, we assessed the intrinsic photoresponse of the exfoliated MoSe_2_ flakes. A photodetector (PD) was fabricated via drop-casting exfoliated MoSe_2_ flakes from a solution on interdigitated Au electrode patterns. Electrical measurements under illuminated and dark conditions were then performed to characterize the photoresponse. A 4 W indoor bulb with a power density of 15 mW cm^–2^ was employed as the light source for all measurements in this work unless otherwise specified (Figures S4 and S5), and the generated light intensity reaching the battery was estimated to be ∼11.3 mW/cm^2^. The *I*–*V* curve of the MoSe_2_ PDs collected under illumination from −3 to 3 V (Figure 2a) shows a current enhancement compared with the current in the dark. This suggests that the exfoliated MoSe_2_ flakes are photoactive within the voltage window, and additional charges can be generated and separated upon exposure to light. To further investigate the characteristics of the photodetector, the electrical current of the device was measured as a function of time. A representative time-dependent photocurrent of the MoSe_2_ photodetector at 1 V is shown in Figure 2b. The device exhibits a significant enhancement in current when it is illuminated by light. The average increase is determined to be 22% (, where *I*_ph_ is the photocurrent and *I*_d_ and *I*_l_ are the electrical currents in the dark and under illumination, respectively). The enhancement in the photocurrent could be attributed to the formation of heterojunctions at the contacts and to the bias assisting the extraction of electrons and holes generated in the semiconductor at increased voltages. We can also consider that the occurrence of “band nesting”, namely the presence of regions in the band structure where conduction and valence bands are parallel to each other in energy, in MoSe_2_ may also contribute to the promotion of self-separation of photogenerated excitons into charges.^48,49^ In the “nesting” region, the electrons and holes will propagate with the same, but opposite, velocities with a short-lived suppression of their direct relaxation.^50^ Furthermore, at 0 V, the current under illumination is 1.43 nA higher than that in the dark, suggesting that intrinsic charge generation occurs without the application of an external voltage.

**Figure 2:**
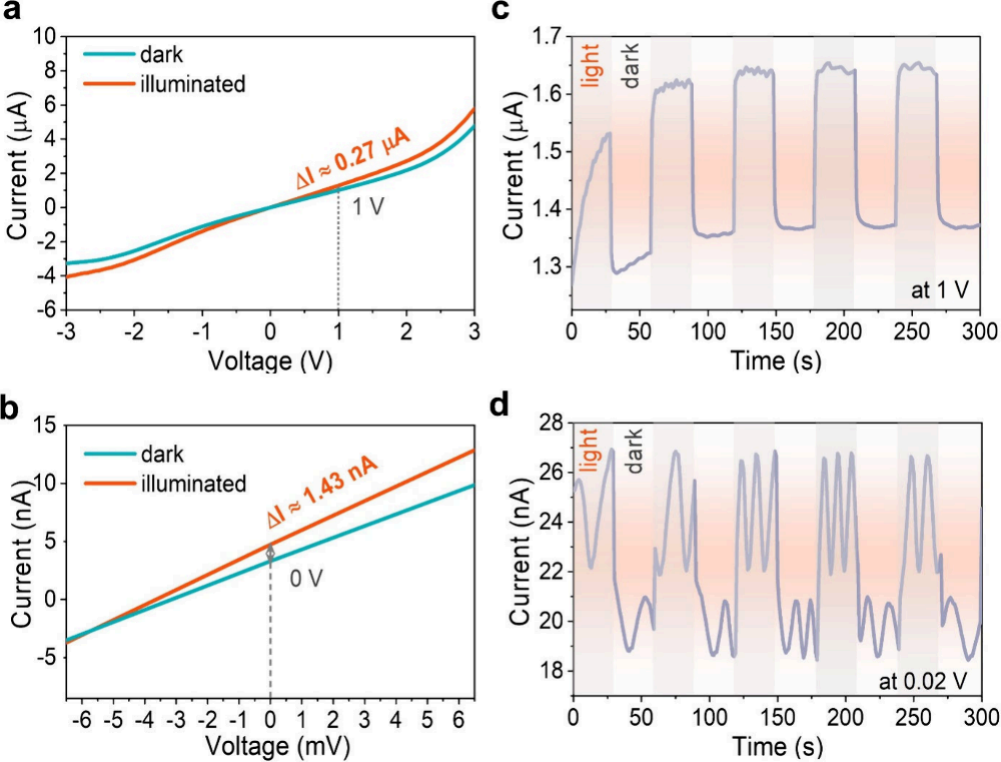
*I–V* curves of a MoSe_2_ photodetector in the dark and under illumination (a) from −3 to 3 V and (b) from −6 to 6 mV. Chopped chronoamperometry measurements of a MoSe_2_ photodetector under dark and illumination at (c) 1 and (d) 0.02 V.

When the external voltage is reduced to 0 V, the fluctuation range of the currents under dark and illumination exhibits significant differences, suggesting that although MoSe_2_ does not exhibit an excellent photocurrent, it still demonstrates photoactivity in the absence of an external electric field (Figure S6). The voltage was then slightly increased to 0.02 V as reported in panels c and d of Figure 2, whereupon MoSe_2_ exhibits a distinct photocurrent, indicating the charge carriers’ separation at 0.02 V. As hypothesized above, the “band nesting” effects in MoSe_2_^51^ may contribute to stimulation of the self-separation of photogenerated excitons into charges in the nesting region in momentum space. Moreover, the relaxation of excitons in thin-layer TMDs from the high-energy C exciton state to lower-energy band-edge optical states (A/B exciton) could also contribute to further suppression of indirect band gap recombinations.^52,53^ These characteristics endow exfoliated MoSe_2_ flakes with the ability to promote self-separated photogenerated charges, which are desirable for establishing a photobattery.^17,19^

Subsequently, a MoSe_2_-based PSIB was fabricated using MoSe_2_ as the photocathode and Na metal foil as the reference and counter electrodes. We used 1 M NaPF_6_ in a 1:1 (v/v) EC/DMC mixture as an electrolyte in the cell. The MoSe_2_ photocathode was made via drop-casting a MoSe_2_-carbon black ink (20 wt %) on carbon paper. The electrochemical performance was evaluated in a windowed coin cell (CR2032) using cyclic voltammetry (CV), galvanostatic charge–discharge (GCD), and electrochemical impedance spectroscopy (EIS) under both dark and illuminated conditions (Figure 3). CV curves of a MoSe_2_ PSIB collected at scan rates from 0.2 to 1.2 mV s^–1^ in a voltage window from 1 mV to 3 V under dark conditions are shown in Figure 3d. In the first reduction scan at 0.2 mV s^–1^, two peaks centered at 0.60 and 0.33 V are observed. The former could be attributed to the intercalation of Na^+^ into MoSe_2_, forming Na_*x*_MoSe_2_, while the latter can be attributed to the conversion of Na_*x*_MoSe_2_ into Na_2_Se and Mo metal.^39,54^ During the first oxidation scan, two peaks appear, at 1.71 and 2.14 V (Figure 3d). The first oxidation peak could be attributed to the recovery of MoSe_2_ through the oxidation of Na_2_Se and Mo (desodiation)^21^ as per the following
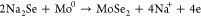
1The CV curves from the second cycle onward present similar peaks, suggesting good reversibility. The two reduction peaks observed in the first cycle disappear, and instead, two new peaks emerge at 1.39 and 2.04 V (at 0.2 mV s^–1^). These peaks can be interpreted in light of the reduction peaks reported in Na–Se batteries with a Se anode.^55^ The first reduction peak at 2.04 V can be ascribed to the reaction from Se to Na_2_Se_*n*_ (4 ≤ *n* ≤ 8), and the second reduction peak at 1.39 V (Figure 3d) corresponds to the further reduction of the sodium polyselenides [Na_2_Se_*n*_ (4 ≤ *n* ≤ 8)] to Na_2_Se_2_, which is an intermediate ahead of the final product (Na_2_Se) after sodiation.^56^ In addition, the oxidation peak located at 0.62 V, arising in the second oxidation scan, could be associated with the onset of the desodiation process and the subsequent partial oxidation of Na_2_Se to Se.^57^ The reformation of MoSe_2_ and the formation of Na_2_Se and polyselendies have been confirmed by operando Raman spectroscopy and supported by operando UV–vis reflectance studies (Figures S13 and S14) and post mortem X-ray photoelectron spectroscopy (XPS) analysis (Figure S13). Overall, the area of the first reduction cycle for the MoSe_2_ PSIB is much larger than that of the second cycle onward, whereas the shape of the oxidation reactions of the first two cycles remains nearly unchanged (Figure 3d). This difference may suggest the formation of the SEI and irreversible reactions occurring during the first reduction process, explaining the low Columbic efficiency of the first GCD cycle (Figure S11a).^58,59^ CV measurements of the MoSe_2_ PSIB under illumination were also performed at the same scan rates as presented in Figure 3e, where an increase in current under illumination compared to the current in the dark occurred. Additionally, a slight decrease in the onset potential in the reduction peaks of the first cycle is detected. CV curves of the MoSe_2_ PSIB under dark and illumination conditions at 1.2 mV s^–1^ are reported in Figure 3f. While enhancements at the extremes of the CV are marginal, we observe that particularly the peak current at the redox events is enhanced under light, with an associated overall 35% increase in the CV area compared to that of the dark case. This suggests that the photogenerated charge carriers enhance the capacity through the redox reactions that they assist, and not through heating, which would increase the kinetics throughout the CV test. The possible oxidation reactions that photogenerated holes can facilitate are as follows (Figures S16 and S17): the valence band holes can repulse the intercalated Na back into the electrolyte via a desodiation process (+0.62 V), thus regenerating MoSe_2_; a competitive reaction at +1.71 V could be a different desodiation process as per 2Na_2_Se + Mo^0^ → MoSe_2_ + 4Na^+^ + 4 e^–^.

**Figure 3:**
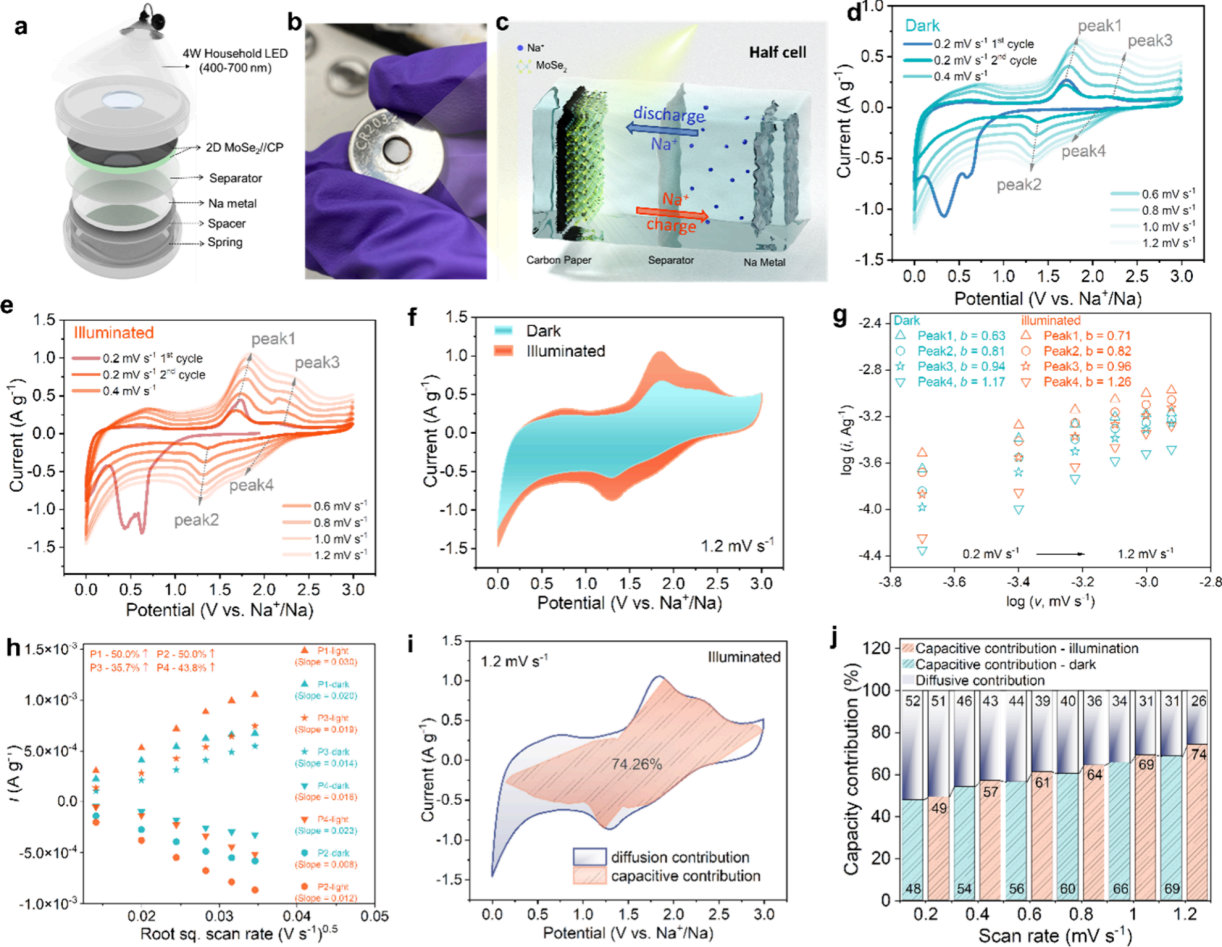
Design of the photo-rechargeable Na-ion battery (PSIB) configuration using exfoliated MoSe_2_ and Na metal. (a–c) Schematic diagrams and an optical photograph of the fabricated SIB. (d and e) CV curves of a MoSe_2_ PSIB from 0.2 to 1.2 mV s^–1^ under dark and illumination conditions, respectively. (f) CV curves of a MoSe_2_ PSIB at 1.2 mV s^–1^ under dark and illumination conditions. (g) Determination of the *b* values of the peak anodic and cathodic currents (from panels d and e) under dark and illumination conditions. (h) Diffusion constant analysis of a MoSe_2_ PSIB under dark and illumination conditions. (i) Capacitive contribution calculation of a MoSe_2_ PSIB under illumination conditions. (j) Bar chart of capacitive contribution calculation at various scan rates under dark and illumination conditions.

The CV curves of a MoSe_2_ PSIB under both dark and illumination conditions at scan rates ranging from 0.2 to 1.0 mV s^–1^ are reported in Figures S7a–f and S8. It is interesting to note that higher scan rates result in larger CV enhancement by area, which suggests that light promotes an increase in charge density, which is more prominent when surface phenomena are dominating. The obtained current response (*i*) under dark or illumination conditions and scan rates (*v*) follows an empirical power law (*i* = *a*ν^*b*^, where *a* and *b* represent the adjustable parameters).^60^ The *b* values were estimated by obtaining the slope of plots of log(*i*) versus log(ν), which provides insights into the charge storage mechanism. When the *b* value is 0.5, a diffusion-controlled process is dominant, and while there is a surface capacitive-controlled process when the *b* value is close to 1. The calculated *b* values (Figure 3g) during the redox process indicate that both surface and diffusion-controlled charge storage were observed in MoSe_2_. To gain deeper insights into the light-enhanced capacity, the contributions from both surface-controlled (capacitive) and diffusion-controlled (faradaic) processes are calculated using the modified power law as they constitute the total response current. As shown in Figure 3h, the slope represents the diffusion constant of each peak under dark or illumination conditions. Under illumination conditions, the diffusion constant increases by ∼50% for peaks 1 and 2, by ∼35% for peak 3, and by ∼43.8% for peak 4. This suggests that light can increase the ion diffusion constants. The CV curves under dark and illumination conditions at 1.2 mV s^–1^ are reported in Figure S9 and Figure 3i, respectively. The contribution from the capacitive process is ∼68% in the dark and increases to ∼74% under illumination, suggesting that MoSe_2_ band gap illumination strongly increases the capacitive contribution. This originated from the increase in charge carrier density upon illumination. The general capacitive contribution from lower to higher scan rates is observed to increase upon augmenting the scan rates due to the surface-mediated capacitive behavior (Figure 3j and Figure S10a–j) under both dark and illuminated conditions.^61^ Overall, the contribution from capacitive behavior in the dark is systematically smaller than that under illumination conditions at each scan rate. As the battery shows good reversibility, the diffusion constant can be appropriately extrapolated from a linear relationship between the peak current (*i*_p_) and diffusion constant (*D*_Na^+^_).

To explore the rate and cycling performance of the MoSe_2_ PSIB under dark and illumination conditions, galvanostatic charge–discharge (GCD) was carried out in the voltage window of 0.2–3.0 V at increasing currents from 60 to 600 mA g^–1^. According to the previous results for MoSe_2_, the MoSe_2_ SIB suffers from poor initial Coulombic efficiency (CE) due to partial irreversible reactions and the massive consumption of Na^+^ toward the formation of SEI in the first discharge cycle,^62,63^ as shown in Figure 4a and Figure S11a. Subsequently, the CE increases from the second cycle.^58,59,64^ Upon exposure to light, the reversible capacities of the first and second cycles increased by ∼5% at 60 mA g^–1^ in both cycles. However, the voltage at which illumination begins to exert an effect, specifically, is at ∼0.95 V for the first cycle and at ∼2.5 V for the second cycle. This is revealed by the positions of the reduction peaks observed in the CV scans for the first two cycles (Figure 3d). Panels a and b of Figure 4 represent the GCD profiles of a MoSe_2_ PSIB at 60 and 600 mA g^–1^ under dark and illumination conditions, respectively (GCD profiles at 150–450 mA g^–1^ are provided in Figure S11b–d). A MoSe_2_ PSIB delivers reversible capacities of 347.1 mAh g^–1^ at 60 mA g^–1^ and 118.8 mAh g^–1^ at 600 mA g^–1^. The corresponding capacity enhancement under light is 19 mAh g^–1^ at 60 mA g^–1^ and 34.8 mAh g^–1^ at 600 mA g^–1^. This suggests that the light induces higher capacity at faster charging rates predominantly in the bias-assisted case. To further verify this, the values of the average capacity enhancement induced by light were calculated from three cycles of GCD profiles at different currents. As shown in Figure 4c, the average reversible capacity enhancement under illumination increases from 6.3% at 60 mA g^–1^ to 29.3% at a specific current of 600 mA g^–1^. This effect is likely due to the increase in the charge carrier density upon illumination. Because the capacity under these conditions is governed by surface phenomena, the effect is more pronounced, as most of the charge carriers generated by light exposure accumulate near the electrode’s surface.^19^ Moreover, a MoSe_2_ PSIB also exhibits a better capacity retention of ∼68% under illumination conditions than under dark conditions (∼64%) at 60 mA g^–1^ (Figure 4d). The cycling stability of the MoSe_2_ SIB at 150 mA g^–1^ under both dark and illumination conditions is reported in Figure 4e. The MoSe_2_ PSIB under light also retains a reversible capacity of 63.3 mAh g^–1^ after 200 cycles, with 42% capacity retention versus a decrease in capacity of 49 mAh g^–1^ in the dark. We could exclude the possibility that the cause of the capacity increase is a heating effect of the battery, because we have measured the temperature of the coin cell through the window, and an increase in temperature was not detected (Figure S18). Additionally, a controlled heating experiment (Figure S19) was conducted, revealing that the battery exhibits a capacity fade after the second cycle when heated. Furthermore, EIS was performed to investigate the charge transfer kinetics of the MoSe_2_ PSIB. As shown in Figure 4f, the results for the MoSe_2_ PSIB under both dark and illumination conditions exhibit two overlapping semicircles in the high-frequency region, followed by an inclined line in the low-frequency region. The corresponding equivalent circuit is presented in Figure S12. The EIS results showed that the MoSe_2_ PSIB exhibits lower resistance, which led to improved charge transfer and ion diffusion under illumination conditions than under dark conditions. Finally, the bare light charging performance was assessed without the application of an external current or voltage. As shown in Figure 4g, the battery was charged only by the light, starting from 0.5 V. Over 2.1 h, the battery displayed 1.0 V, and after 46 h, the battery was at 1.68 V. This evidence suggests that light facilitates the charging of the NaMoSe_2_ cathode, which involves desodiation of MoSe_2_. The estimated solar to energy conversion efficiency hence equals ∼0.19%. To discharge the battery, Na^+^ ions are deintercalated, and the current is matched by electrons from the Na anode. The fate of the photogenerated electrons could be as follows. Some of the electrons can recombine with the holes or could participate in reductive reactions involving Na^+^ that can lead to the formation of SEI on the cathode. The latter could be also a parasitic reaction taking place with the electrolyte or Na^+^ ions having been deintercalated by the holes. It could be a reduction reaction of MoSe_2_ with Na^+^ forming Na_2_Se and Mo, which diffuse into the electrolyte or form an SEI that cannot be accessed in further cycles.

**Figure 4:**
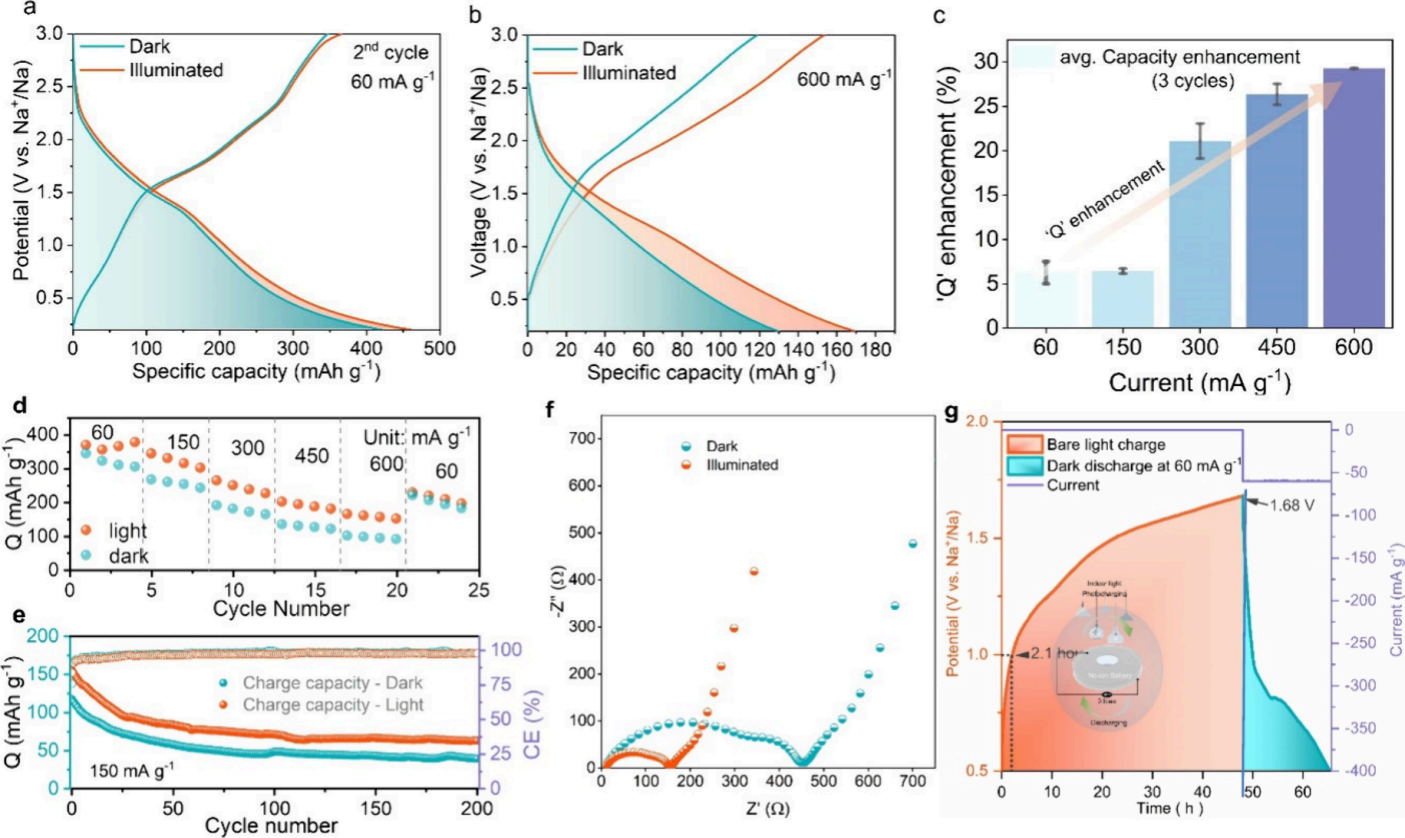
GCD profiles of the MoSe_2_ PSIB for the second cycle at (a) 60 and (b) 600 mA g^–1^. (c) Average capacity enhancement (%*Q*) of a MoSe_2_ PSIB at different specific currents. (d) Rate performance of a MoSe_2_ PSIB. (e) Cycling stability measurement of a MoSe_2_ PSIB. (f) Nyquist plots taken through EIS of a MoSe_2_ PSIB. (g) Bare light charging of a MoSe_2_ PSIB.

This work has demonstrated a PSIB, which exploits the strong light absorption of MoSe_2_ crystals used as cathode material. The MoSe_2_ PSIB can be charged by a 4 W LED without applying an external voltage or current, by photon-driven deintercalation of Na^+^ from the cathode. The light also contributes to increasing the capacity from 6% at 60 mA g^–1^ to 29% at a higher specific current of 600 mA g^–1^, suggesting that the capacity increase is more prominent at faster charge rates. This not only is the effect of the additional photocurrent but also appears to be a consequence of the overall increase in charge transfer kinetics and ion diffusion under illumination. This work paves the way for the development of batteries with an integrated energy-harvesting system for future energy autonomous systems.

## Supporting Information Available

Experimental details; STEM and SEM images of MoSe_2_ flakes; XPS analysis of exfoliated MoSe_2_; digital photographs of a MoSe_2_ drop-casted photodetector device; PL spectrum of a household LED lamp; CV curves of a MoSe_2_ PSIB under dark and illumination conditions; mechanism of the multistep transformation of sodium polyselenides in a Na–Se battery; diffusion constant calculations; charge–discharge curves under light and dark conditions; EIS equivalent circuit and its parameters; operando Raman measurements, operando UV–vis reflectance spectra, and ex situ XPS analysis; energy band level diagram of MoSe_2_ during the photoassisted charging process; schematic representation of the photocharging process; light exposure of a PSIB and the temperature effect; charge–discharge analysis of PSIB at 50 °C; and work function measurement (PDF)
