# Supplementary material for: Photo-Rechargeable Sodium-Ion Batteries with a Two-Dimensional MoSe2 Crystal Cathode
Source: Nano Lett. 2025 Jan 15;25(5):1775–82. doi: 10.1021/acs.nanolett.4c03471 (PMC11803744; doi:10.1021/acs.nanolett.4c03471)
Supplement: Supplementary file 1 — nl4c03471_si_001.pdf [file nl4c03471_si_001.pdf]

Supplementary Information

Photo-Rechargeable Sodium-Ion Batteries with a 2D $MoSe_2$ Crystals -Cathodes

Gang Cheng[a, #], *Zhenyu Guo*[b, #], *Nagaraju Goli*[a], *Filip M Podjaski*[c], *Kaitian Zheng*[b], *Jinglin Jiang*[b], *Sami Ramadan*[a], *Gwilherm Kerherve*[a], *Stefano Tagliaferri*[a], *Mauro Och*[a], *Norbert Klein*[a], *Mattia Cattelan*[d], *Stefano Agnoli*[d], *Maria-Magdalena Titirici*[b], *Cecilia Mattevi*[*, a]

[a] Department of Materials, Imperial College London, London, SW7 2AZ, UK

[b] Department of Chemical Engineering, Imperial College London, London, SW7 2AZ, UK

[c] Department of Chemistry and Centre for Processable Electronics, Imperial College London, 80 Wood Lane, London W12 7TA, UK

[d] Department of Chemistry and INSTM Padua research Unit, University of Padua, Via Marzolo 1 35131, Padova Italy

[#] These authors contributed equally to this work

*Corresponding authors:

Cecilia Mattevi (c.mattevi@imperial.ac.uk)

Gang Cheng (gc1417@imperial.ac.uk)

Experimental section

Materials

The $MoSe_2$ powder was purchased from Alfa Aesar (99.9%). The carbon paper (TGP-H-60) was purchased from Tory Ltd. (Japan). The isopropanol solvent (IPA) was purchased from VWR Ltd. Sodium metal (Alfa Aesar, 10342 Sodium ingot, 99.8%, metals basis) was purchased from Merck. The $NaPF_6$ salt (99.0 %), EC (99.9 %), DMC (99.9 %) solvents, and quartz glass window were purchased from Guangdong Canrd New Energy Technology Ltd (China). Expoxy glue was purchased from Araldite. Glassy fiber (grade A) was bought from Whatman. Carbon black was bought from Ketjenblack. Coin cells (CR2032) and copper foil were purchased from both Pikem Ltd (MTI UK distributor) and Guangdong Canrd New Energy Technology Ltd (China). The 4W LED light source was purchased from Philips model GU10.

The process for $MoSe_2$ exfoliation

The exfoliated $MoSe_2$ flakes were prepared by dispersing 244 mg of $MoSe_2$ powder into 20 ml of 30% v.% IPA solvents with Di-water through sonication (Fisherbrand Model 705 sonic dismembrator)[1]. The parameters for sonication were 40 amplitude with 5s of pulse-ON time and pulse-OFF time respectively. The dispersion after sonication was left for 24 hours to allow the unexfoliated material to sediment.

Electrode preparation

The stable supernatant of the exfoliated $MoSe_2$ flakes dispersion was used for drop-casting. Before drop-casting, the average concentration was measured by drying 1 ml of the dispersion. Then 20% of carbon black was added to the dispersion with known concentration and sonicated for 10mins. The dispersion was then drop-coated onto a carbon paper substrate (1 cm in

diameter) on a hot plate at 80 ^{o}C. Followed by drop casting, the electrode was dried in a vacuum oven at 80 ^{o}C overnight before importing into a glovebox.

Electrochemistry

An argon-filled glovebox (H_2O < 0.5 ppm, O_2 < 0.5 ppm, mBraun) was used to assemble all CR2032 coin cells. A piece of Na metal (12 mm in diameter) was used as both a reference and a counter electrode. Glassy fiber (grade A) soaked with 100 µL 1M $NaPF_6$ in EC: DMC (1:1 in vol.) was used as the separator. The configuration of the coin cell composed of exfoliated $MoSe_2$ on carbon paper as cathode, 1M $NaPF_6$ in EC: DMC (1:1 in vol) as electrode and Na metal as anode. All coin cells are assembled in the same manner to minimize electrochemistry differences in performance (e.g., 0.6-0.7 mg cm^{-2} of mass loading, same pressure of 70 psi). All the coin cells were discharged-charged in a potential window of 0.2 – 3.0 V (V vs. Na^+/Na). Galvanostatic charge and discharge, rate performance, and long cycling were performed on a LAND CT2001A battery testing system. Cyclic voltammetry (CV) and electrochemical impedance spectroscopy (EIS) were both performed on either a Palmsens potentiostat system or a Biologic potentiostat system.

Electrochemical measurements under light

The galvanostatic charge and discharge, CV, and EIS measurements were performed using coin cells with a 6mm quartz window accessible for light. The light was always open during those tests. The light source is only a 4W LED light. Its power density was measured and averaged by a power meter (Gentec UNO power meter) as 11.3 mW cm^{-2}. The measurement distance between the light source and the power meter was fixed at a vertical distance of 10cm equal to the light source to the coin cell.

Structural characterization and analysis

The morphology of the exfoliated $MoSe_2$ was visualized by scanning electron microscopy (SEM, Zeiss Auriga) and transmission electron microscopy (TEM JEOL STEM 2100F). X-ray diffraction (XRD) was carried out by an X'Pert PRO PANalytical (40 mA and 40 kV for power settings). Raman spectroscopy was performed on a Renishaw Raman machine using a 532 nm laser. XPS was performed by a Thermo Fisher K-Alpha$^+$ XPS facility, and all data were fitted using the Advantage software. K-alpha. AFM was conducted on an Asylum MFP-3d microscope equipped with a PtIr-coated Silicon probe (Bruker SCM-PIT-V2). UV-VIS was performed using a PerkinElmer Lambda 25 UV-VIS spectrometer in 1 cm path quartz cuvettes.

Photodetector fabrication and electrical measurements:

Fabrication and electrical measurements of MoSe2 photodetector structures: photoresist was spun on SiO2/Si substrates and the electrodes structures were patterned using optical lithography, then 5nm/50 nm Ti/Au was deposited on the sample using Magnetron Sputtering, followed by lift-off process in acetone to remove the photoresist. Two-probe measurements were performed to measure the electrical resistance of MoSe2 with and without light. All electrical measurements were performed at room temperature using B1500 Semiconductor Analyzer.

Electrode preparation for *ex-situ* XPS

The electrodes for ex-situ XPS measurement are prepared by pre-cycling at 60 mA g^{-1} for 1 cycle between 0.2 – 3.0 V before dissembling in an argon-filled glovebox (H_2O < 0.5 ppm, O_2 < 0.5 ppm, mBraun). The ex-situ XPS analysis were also carried out on dark/photo-charged cell at 1.68V. The electrodes were carefully washed with DMC solvent to remove the residue $NaPF_6$ salt that may contaminate the electrode. Following the washing process, the electrode

was transferred to the antechamber of the glovebox and leave it in a vacuum environment without heating for 6 hours to fully remove the residue DMC. When it was dried, a vacuum vessel was used to safely transfer the electrode from the glovebox to the XPS chamber to ensure a minimal level of oxygen exposure and surface contamination.

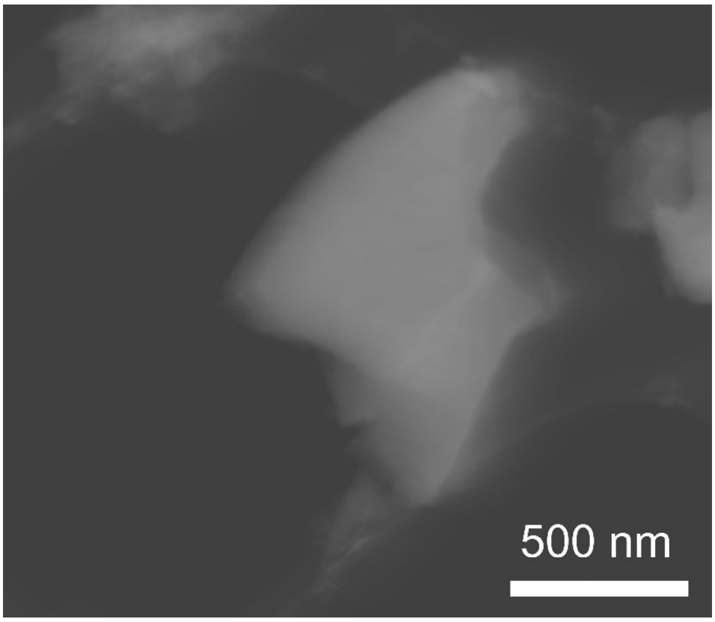

Figure S1 HAADF-STEM image of an exfoliated $MoSe_2$ flake.

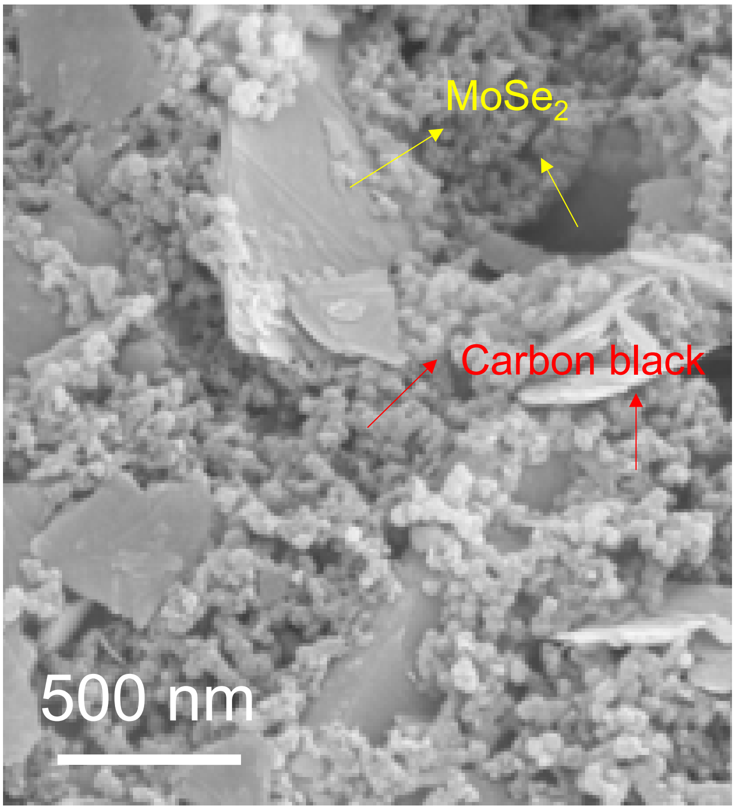

Figure S2 Magnified SEM image of the drop casted $MoSe_2$ flakes and carbon black on carbon paper.

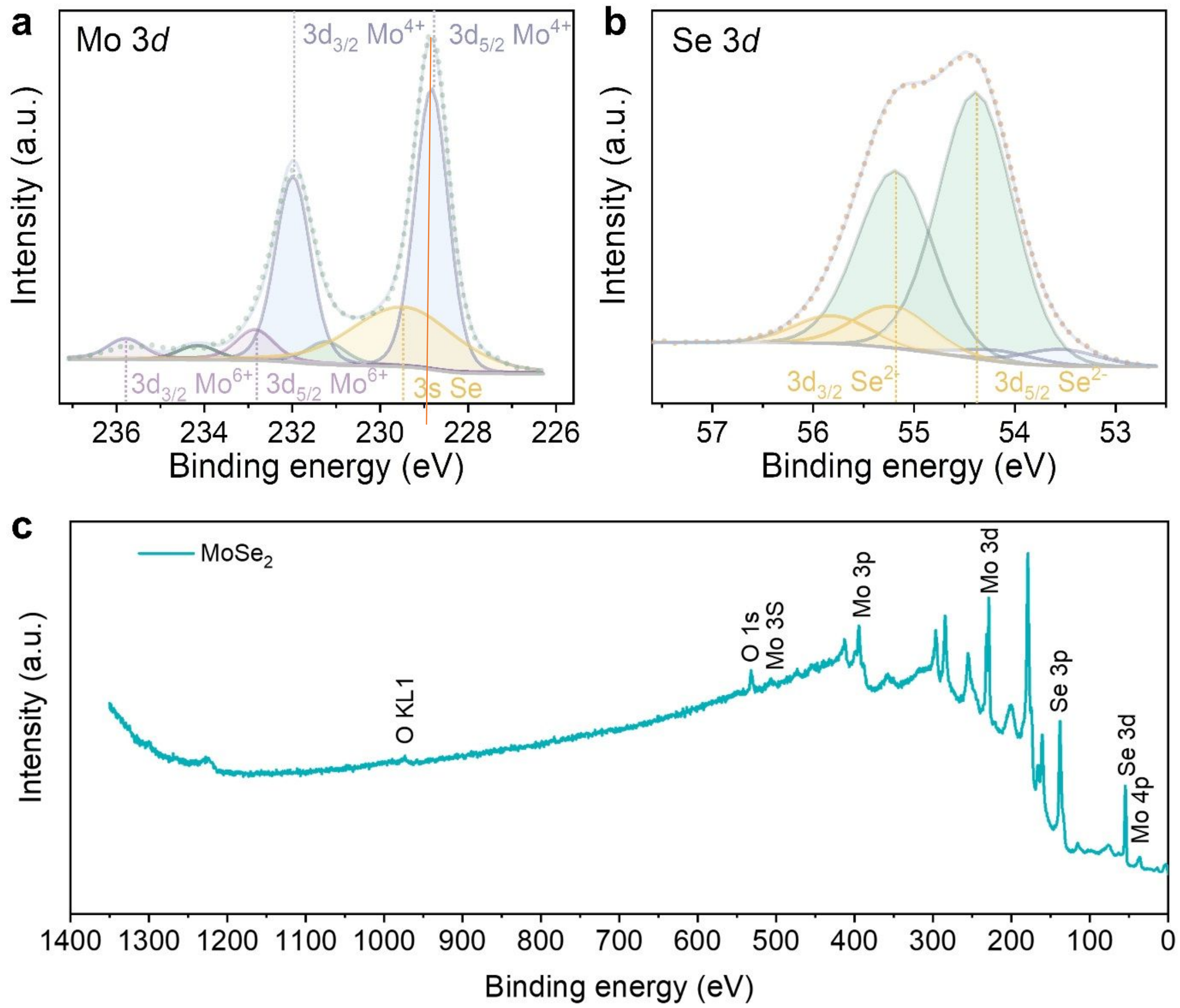

Figure S3 XPS core levels of Mo 3d and Se 2p and a survey (c) of the as-prepared $MoSe_2$ flakes.

The chemical composition of the $MoSe_2$ flake was studied by X-ray photoelectron spectroscopy (XPS) as shown in Figure S3. Mo $3d_{5/2}$ and $3d_{3/2}$ core levels were centered at 228.80 and 231.96 eV respectively as expected for Mo^{4+} in the 2H phase.[2] A small amount of MoO_3 is detected as evidenced by the two components centered (purple shading) at 232.3 and 235.78 eV are attributed to Mo $3d_{5/2}$ and $3d_{3/2}$ of Mo^{6+}.[3] Selenium is predominantly found as Se^{2-} which is expected in the 2H - $MoSe_2$ (Se $3d_{5/2}$ and Se $3d_{3/2}$ core levels centered at 54.37 and 55.17 eV) . While a small amount of Se in a higher oxidation state is evidenced by the Se $3d_{5/2}$ and Se $3d_{3/2}$ core levels are centered at 55.21 and 55.81 eV[4] respectively.

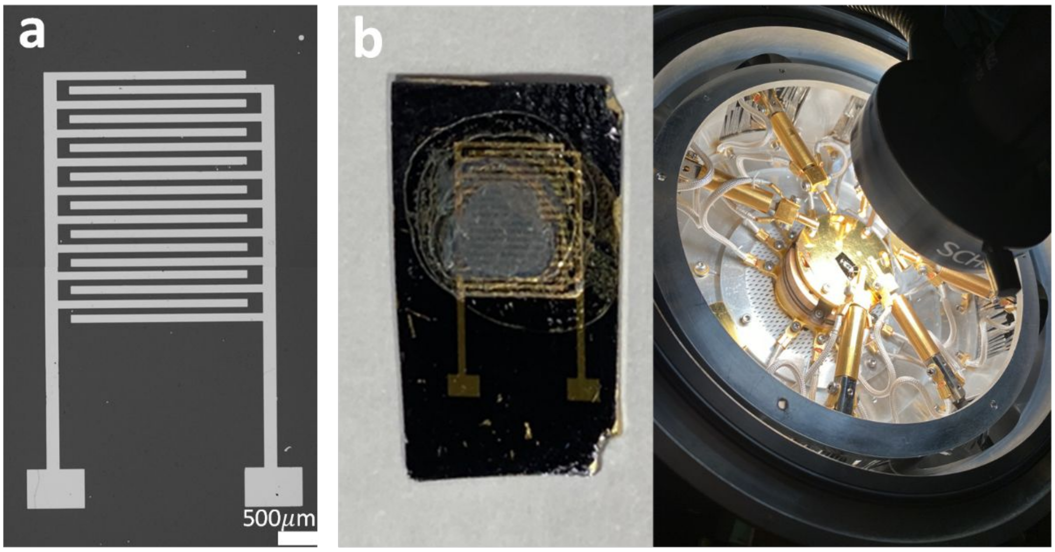

Figure S4 Digital photograph of (a) blank IDE (b) IDE drop-casted with exfoliated $MoSe_2$ flakes under measurement using probe station

Exfoliated $MoSe_2$ flake dispersion was drop-cast on gold interdigitated electrodes (IDE) and tested using a probe station.

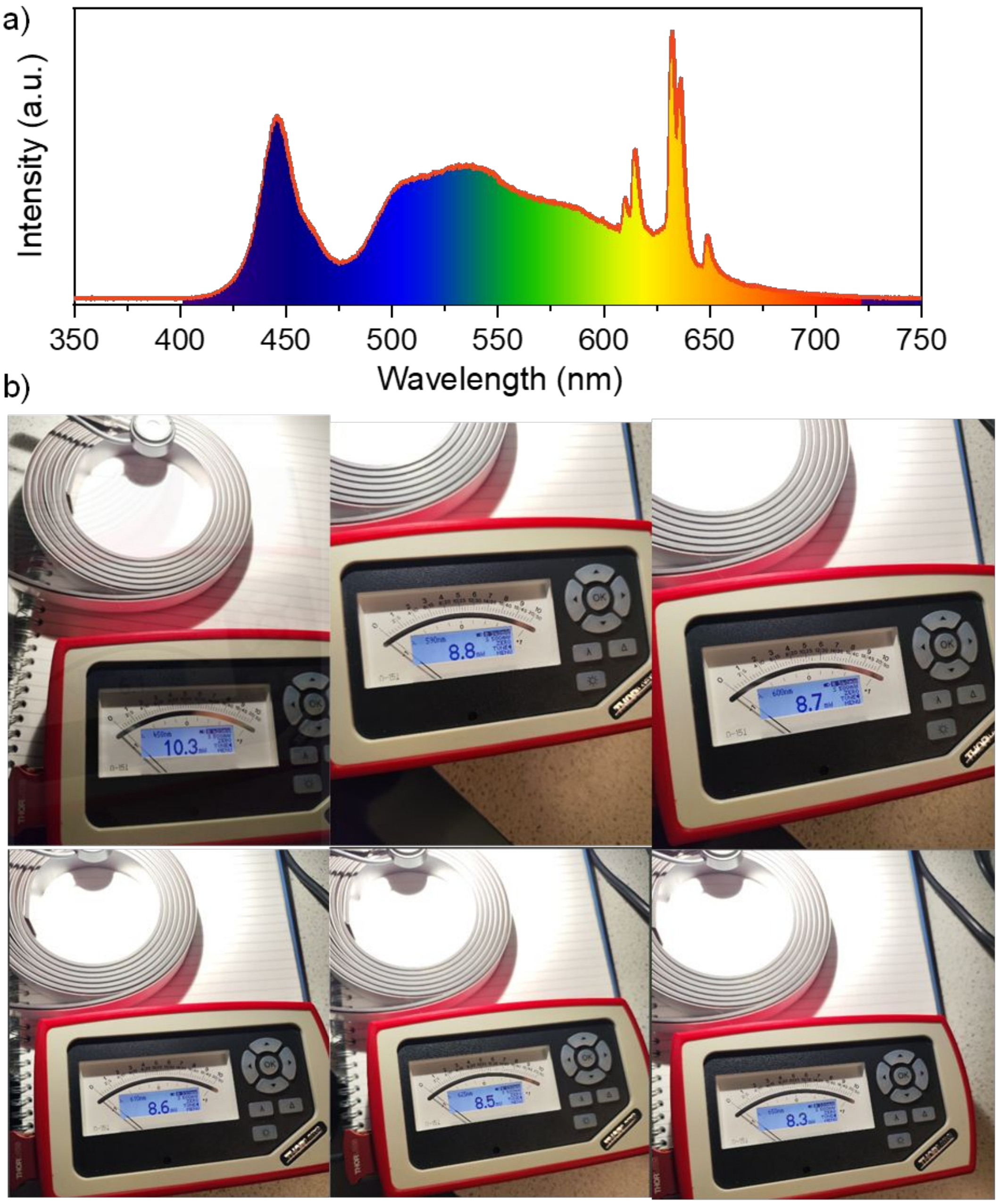

Figure S5. *The PL-spectrum of the household LED lamp used in this work (The spectrum ranges from 400 nm to 700 nm) and their power intensities (10.3-8.3 mW) under different wavelengths of 450-650 nm, respectively.*

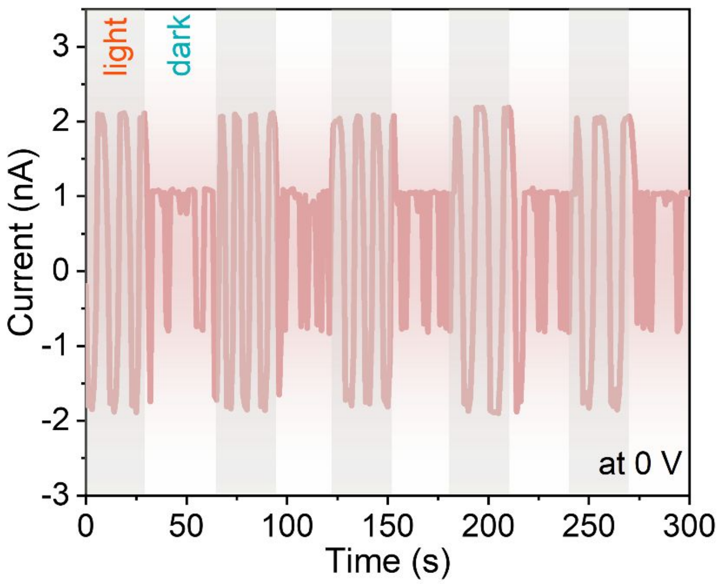

Figure S6 Chronoamperometry of a $MoSe_2$ PDs at 0 V.

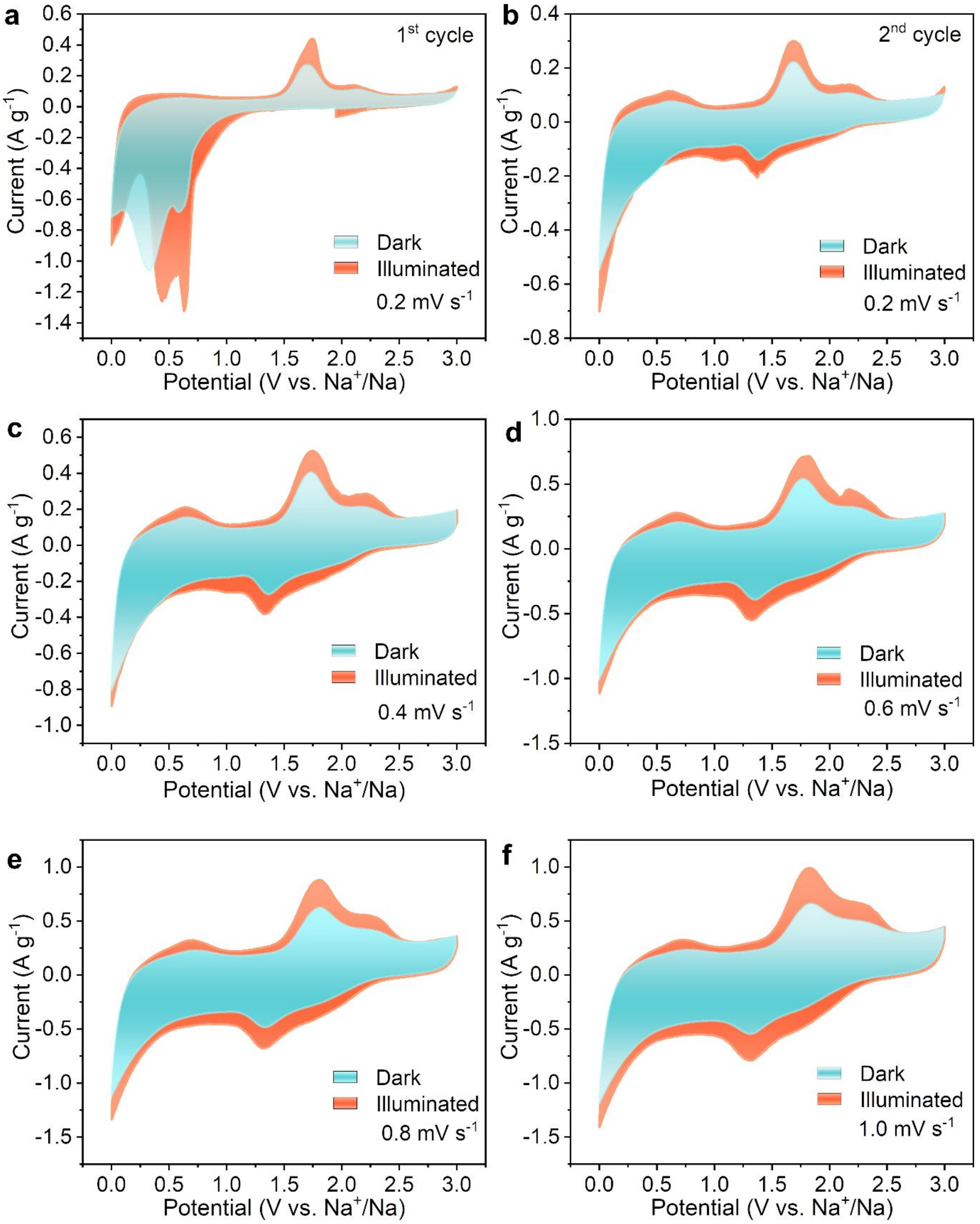

Figure S7 CV curves of $MoSe_2$-PSIB at lower scan rates of 0.2 to 1.0 mV s^{-1} under dark and illuminated conditions.

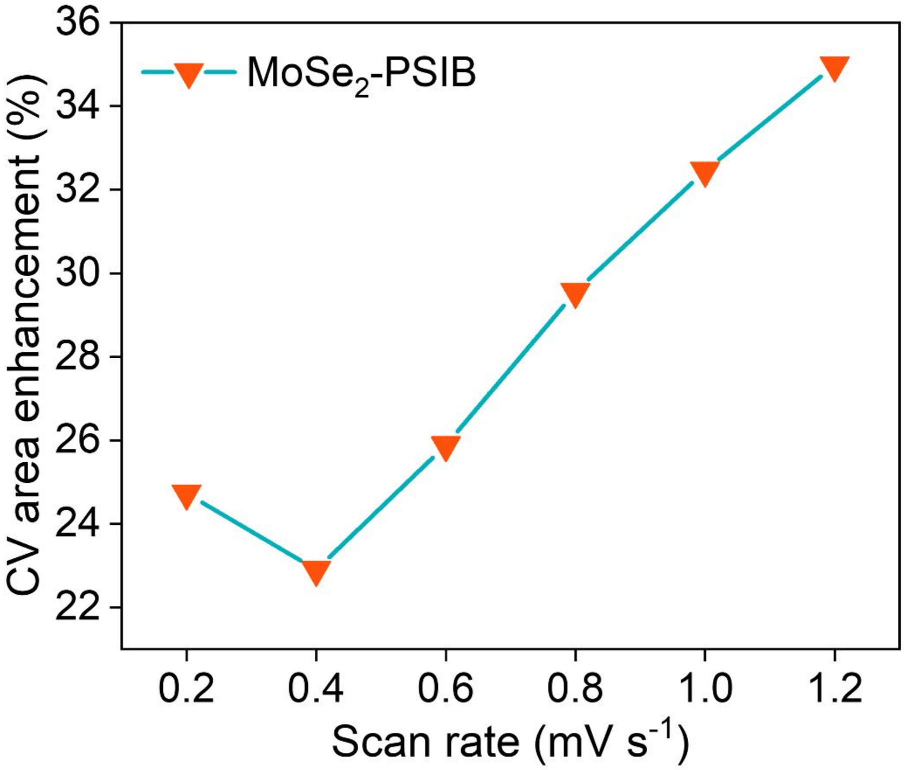

Figure S8 Plot of CV area enhancement of a $MoSe_2$-PSIB under dark at 0.2 to 1.2 mV s^{-1}.

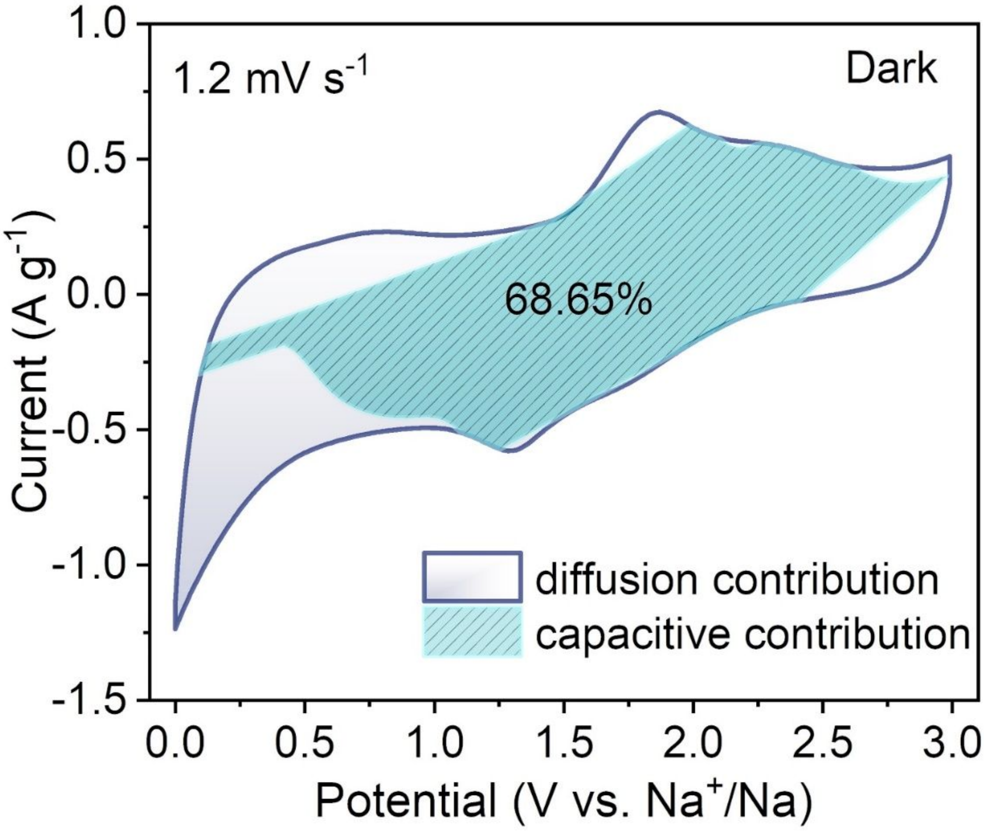

Figure S9 Capacitive contribution calculation of $MoSe_2$-PSIB under dark.

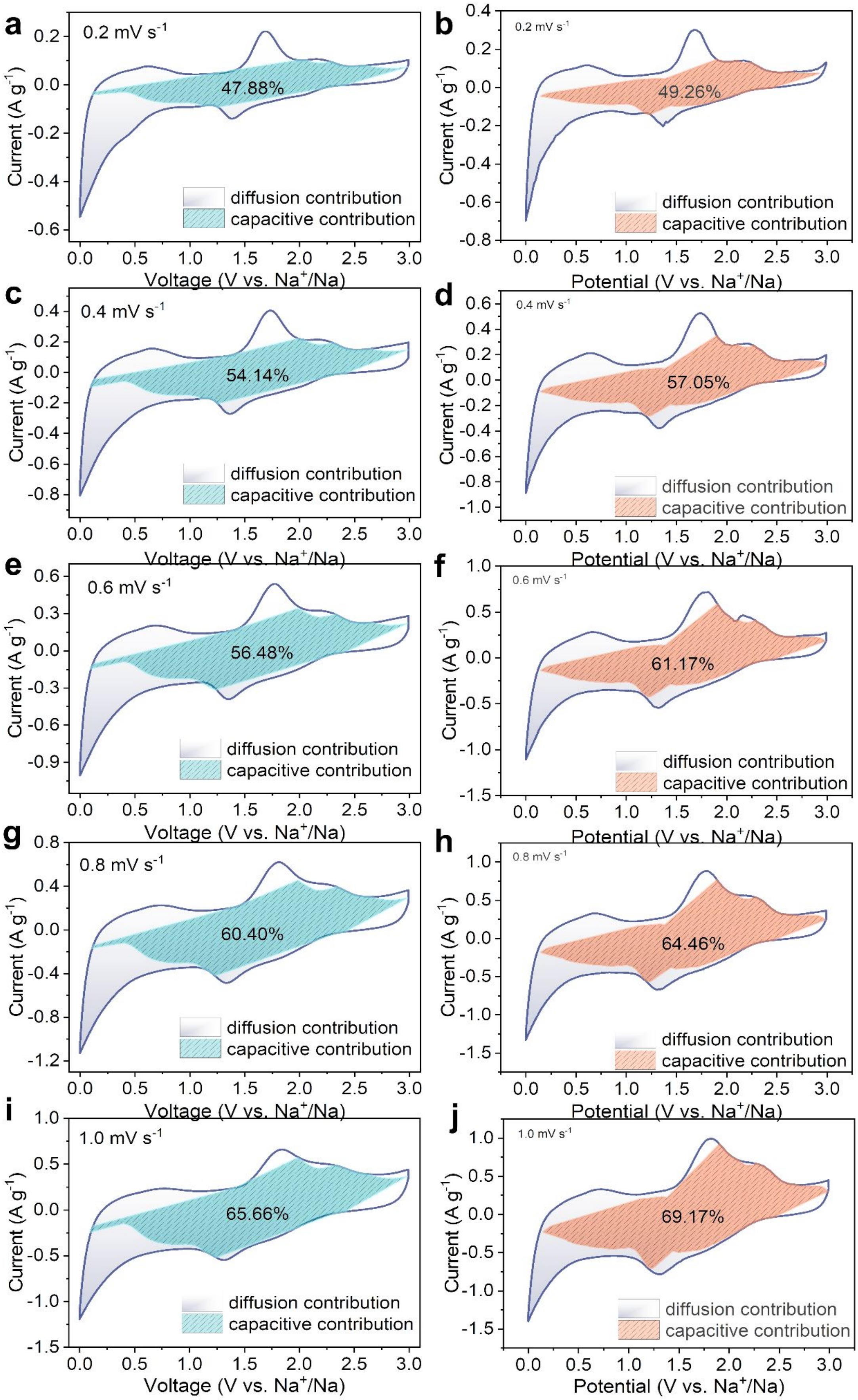
a
0.2 mV s⁻¹
47.88%
diffusion contribution
capacitive contribution
Current (A g⁻¹)
Voltage (V vs. Na⁺/Na)
b
0.2 mV s⁻¹
49.26%
diffusion contribution
capacitive contribution
Current (A g⁻¹)
Potential (V vs. Na⁺/Na)
c
0.4 mV s⁻¹
54.14%
diffusion contribution
capacitive contribution
Current (A g⁻¹)
Voltage (V vs. Na⁺/Na)
d
0.4 mV s⁻¹
57.05%
diffusion contribution
capacitive contribution
Current (A g⁻¹)
Potential (V vs. Na⁺/Na)
e
0.6 mV s⁻¹
56.48%
diffusion contribution
capacitive contribution
Current (A g⁻¹)
Voltage (V vs. Na⁺/Na)
f
0.6 mV s⁻¹
61.17%
diffusion contribution
capacitive contribution
Current (A g⁻¹)
Potential (V vs. Na⁺/Na)
g
0.8 mV s⁻¹
60.40%
diffusion contribution
capacitive contribution
Current (A g⁻¹)
Voltage (V vs. Na⁺/Na)
h
0.8 mV s⁻¹
64.46%
diffusion contribution
capacitive contribution
Current (A g⁻¹)
Potential (V vs. Na⁺/Na)
i
1.0 mV s⁻¹
65.66%
diffusion contribution
capacitive contribution
Current (A g⁻¹)
Voltage (V vs. Na⁺/Na)
j
1.0 mV s⁻¹
69.17%
diffusion contribution
capacitive contribution
Current (A g⁻¹)
Potential (V vs. Na⁺/Na)

Figure S10 Capacitive contribution calculations at lower scan rates under dark (left side plots) and illumination (right side plots), respectively.

Multistep transformation of sodium polyselenides in a Na-Se battery

The multistep transformation of sodium polyselenides in a Na-Se battery can be generally described as the following reactions.[5] The reaction from solid phase Se_8 to liquid phase of long chain sodium polyselenides at 1.7 V. The reactions occur in this process can be described as,

$$\mathrm{nSe} + 2\mathrm{Na}^{+} + 2\mathrm{e}^{-} \rightarrow \mathrm{Na_2Se_n}\ (4 \le \mathrm{n} \le 8) \quad (1)$$

The long chain Na_2Se_n is gradually reduced to Na_2Se_2 at 1.33 V. The reactions are all happen in liquid phase which can be described as,

$$\mathrm{Na_2Se_n} + 2(\mathrm{n}-1)\mathrm{Na}^{+} + 2(\mathrm{n}-1)\mathrm{e}^{-} \rightarrow \mathrm{nNa_2Se_2} \quad (2)$$

Finally, the Na_2Se_2 is converted to Na_2Se and can be described as,

$$\mathrm{Na_2Se_2} + 2\mathrm{Na}^{+} + 2\mathrm{e}^{-} \rightarrow 2\mathrm{Na_2Se} \quad (3)$$

Quantitative evaluation of the effects from capacitive (k_1v) and diffusion-controlled (k_2 $v^{0.5}$) processes

To quantitatively evaluate the effects from capacitive (k_1v) and diffusion-controlled ($k_2v^{0.5}$) processes equation (4) is adopted:

$$i = k_1v + k_2v^{0.5} \quad (4)$$

The cumulative pseudo-capacitance resulted from the capacitive contribution can be calculated by fitting the constants $k_1$and k_2 at different potentials.[6]

Diffusion constant calculation

The diffusion equation can be appropriately solved with a linear relationship between peak current (i_p) and diffusion constant (D_{Na^+}), as given in equation (5),

$$i_p = 0.4463F\left(\frac{F}{RT}\right)^{1/2} C^* v^{1/2} A {D_{Na^+}}^{1/2} \quad (4)$$

where i_p represents the peak current, F is the Faraday constant, $C^*$is the initial concentration in mol cm^{-3}, v is the scan rate in V s^{-1}, A is the area of the electrode in cm^2 and D_{Na^+} is the diffusion constant of Na^+. The equation can be further simplified as equation (6),

$$i_p = D_{\propto Na^+} v^{1/2} \quad (5)$$

where $D_{\propto Na^+} = 0.4463F(\frac{F}{RT})^{1/2}C^*AD_{Na^+}{}^{1/2}$ is a constant proportional to $D_{Na^+}{}^{1/2}$. By estimating the slope of $v^{1/2}$ against i_p under both dark and illuminated conditions, the relative variation in diffusion constants can be compared.

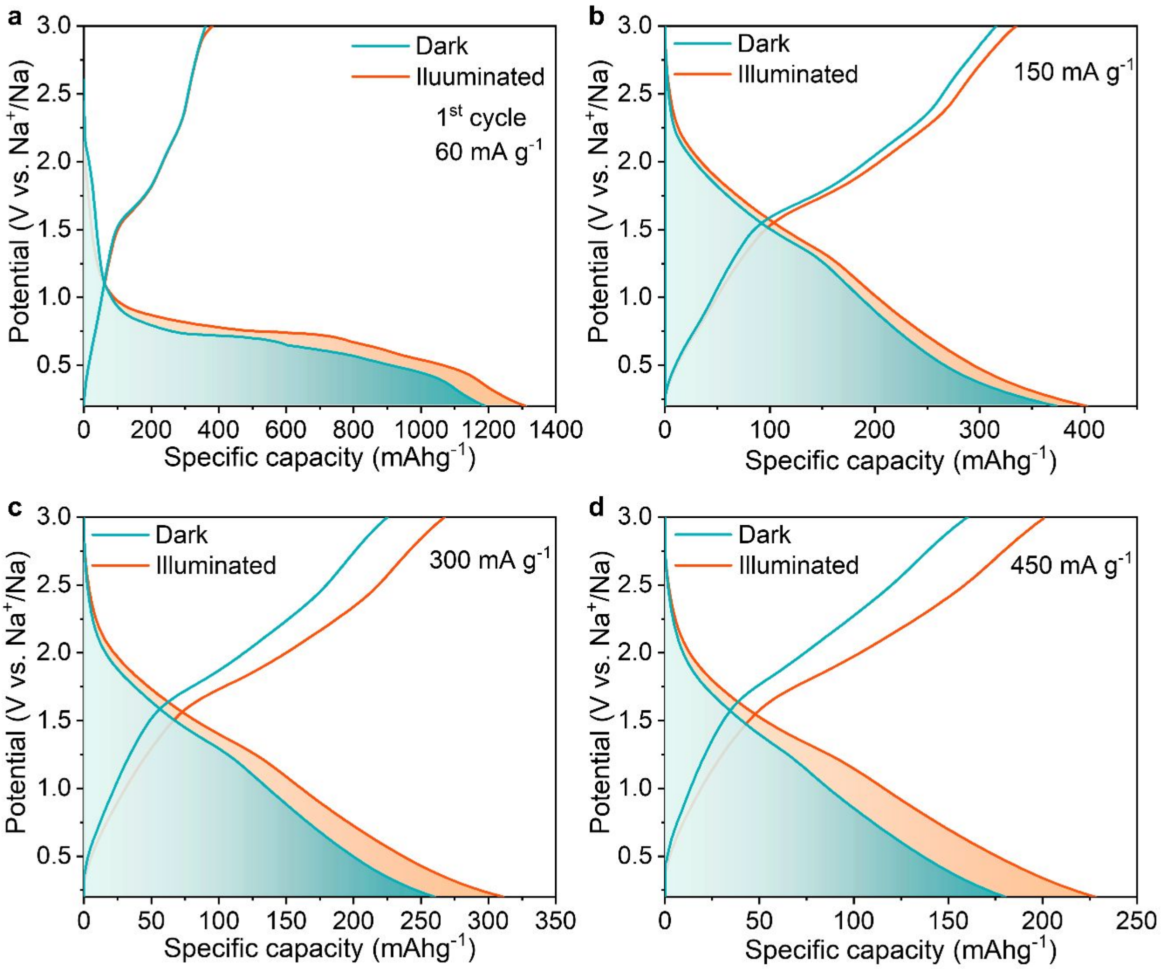

Figure S11 Galvanostatic charge discharge curves at (a) 60 mA g^{-1} for the 1st cycle, (b) 150 mA g^{-1}, (c) 300 mA g^{-1}, (d) 450 mA g^{-1} under dark and illumination.

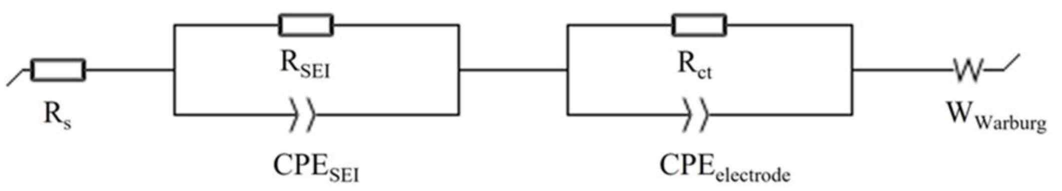

Ω	R_s	R_{SEI}	R_{ct}	$W_{warburg}$
Dark	8.88	231.1	197.8	105.9
Illuminated	11.8	41.1	95.9	62.1
Reduced	33.1% ↑	82.2% ↓	51.5% ↓	41.4% ↓

Figure S12 The equivalent circuits of the impedance spectra shown in Figure 4f.

From the EIS, R_s represents the ohmic internal contact resistance, R_{SEI} is the resistance of the SEI film and ionic resistance of the surface morphology, R_{ct} is the charge transfer resistance, and $W_{warburg}$ relates to Warburg resistance indicating the diffusion of Na^+.[7] $MoSe_2$-PSIB exhibits lower resistance involving charge transfer and ion diffusion under illumination compared to dark. The R_{SEI}, R_{ct} and $W_{warburg}$, decrease of ~82%, ~51% and ~41% respectively. The significant decreases in R_{SEI} and R_{ct} suggest that the light reduces the SEI/ionic resistance within the material including its SEI, and on the surface through charge transfer resistance.

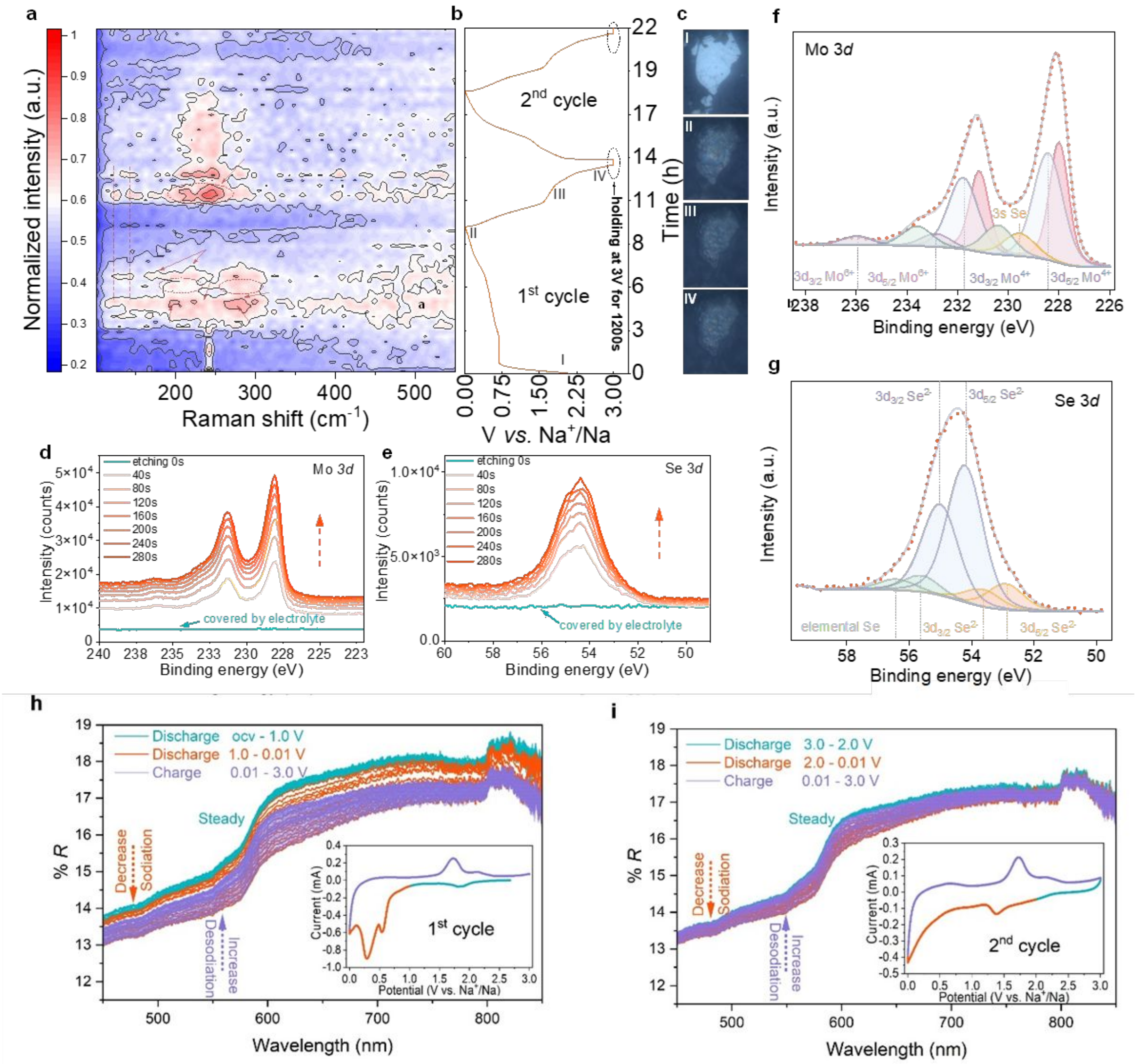

Figure S13: (a) Operando Raman spectroscopy 2D contour plot of a $MoSe_2$-PSIB; (b) corresponding GCD profiles of the first two cycles; and (c) optical images captured by the integrated microscopy during the operando Raman measurement; XPS depth profiles of the $MoSe_2$-PSIB at 3 V; XPS depth profile spectra of (d)Mo 3d5/2 and 3d3/2 and Se 3d; XPS spectra of (f)Mo 3d5/2 and 3d3/2 and (g)Se 3d of as a function of the sputtering time of a $MoSe_2$ electrode after cycling at 3.0 V. Operando UV-Vis reflectance spectra of the $MoSe_2$-SIB for the (h) first cycle and (i) second cycle.

To further understand the Na^+ storage mechanism in $MoSe_2$-PSIB and the role of illumination, operando characterization techniques were employed. The operando Raman 2D contour of $MoSe_2$-SIB for the first two charge/discharge cycles is reported in Figure S13a-c. A prominent A_{1g} mode of 2H-$MoSe_2$ at ~242.1 cm^{-1} is observed at open circuit voltage (OCV)

before cycling. As the first discharge begins, the A_{1g} band gradually diminishes and disappears at ca. 0.7 V where the intercalation of Na ions starts. New vibration modes at 120.8 cm^{-1} (J1), 148.3 cm^{-1} (J2), 198.3 cm^{-1}(J3), 235.9 cm^{-1} (A_{1g}) and 284.6 cm^{-1} (E_{2g}^{1}) appear at ca 0.6 V. These peaks are characteristics of the 1T' phase of $MoSe_2$.[8] This phase transformation is an unequivocable fingerprint of the intercalation of Na between the layers of $MoSe_2$.[9] Additionally, the intense peak at ~277.1 cm^{-1} can be ascribed to the E_{2g}^{1} mode, indicating shear displacements between rigid planes of chalcogenide layers and transition metal layers.[10] This suggests a significant lattice expansion and distortion resulted from Na^{+} intercalation[11] . As the potential drops from 0.6 to 0.3 V, the characteristic peaks (J1, J2, J3) of 1T'-$MoSe_2$ disappear, indicating the start of the conversion reaction from Na_xMoSe_2 to Na_2Se and Mo metal. [11] Meanwhile, two broad bands ranging from 182.1 to 228.4 cm^{-1} and 263.4 to 306.9 cm^{-1} appear. These Raman modes can be associated with reaction intermediates such as Na_2Se_x (x=3-8) and polyselenides such as Se_8 rings. At ca 0.3 V, two new peaks at 165 and 220.8 cm^{-1} are identified, and these are likely to be originated from the formation of Na_2Se which is typically expected to be the final product of the conversion reaction in $MoSe_2$ SIB in the first discharge.[5, 12, 13] Light could partially facilitate polyselenides redox reactions[14], and thus this could potentially increase the reversibility of $MoSe_2$ SIB. In the first charge, no obvious peak is observed before 1.5 V. Interestingly, the characteristic Raman modes of 1T-$MoSe_2$ reappear at 1.7 V indicating that $MoSe_2$ is reformed. As the desodiation process continues, the Raman modes of the 1T' phase of $MoSe_2$ disappear again at 3.0 V as the majority of Na^{+} have been extracted from $MoSe_2$ with the completion of desodiation. However, weak peaks around 240 cm^{-1} can be observed, which could possibly be ascribed to the 2H-$MoSe_2$ enriched in Selenium.[15] From the second cycle onwards, weak J1 and J2 peaks are still observed upon the intercalation of Na^{+}, suggesting that 1T' $MoSe_2$ is partially recovered after the first cycle. Stronger peaks are mainly concentrated in the range of 200 to 280 cm^{-1}, and the characteristic peak for Na_2Se is observed

again when the potential is close to 0 V. This indicates that the battery is governed by the transformation processes of sodium polyselenides after the second cycle, like those in Na-Se batteries and $MoSe_2$ is not reformed. To further verify the final product at the end of the first cycle (3 V), ex situ depth profile using XPS was conducted. Figure S13d, e represents the depth profiles of Mo 3d, Se 3d. The presence of Mo and Se was detected only upon in situ sputtering of the surface electrode using Ar to remove the deposited electrolyte (Figure S13d-g and Figure S14a-c). The XPS analysis of the Mo $3d_{3/2}$ and 3d $_{5/2}$ core levels at ~ 231 eV and ~228.0 eV A (Figure s13d and f) prominent doublet of Mo $3d_{3/2}$ and 3d $_{5/2}$ at 228.42 eV and 231.77 eV can be ascribed as a convolution of two different components: 1T $MoSe_2$ (~228.0 eV) and a Na intercalated 1T-$MoSe_2$ at 228.5eV[16, 17] (Figure S13f).[18] The Na intercalated in the 1T $MoSe_2$ phase is in agreement with the observed presence of Na in the depth profile for Na 1s as shown in Figure S14c. Additional two almost negligible doublets of Mo $3d_{3/2}$ and 3d $_{5/2}$ at 232.8 eV, 236 eV and 230.4 eV, 233.6 eV respectively can be observed, and they can be attributed to the presence of Mo^{6+} and Mo^{4+} in MoOx (Figure S13f).[13, 19] It is worth noting that there is the presence of Se 3s at ~230 eV (Figure S13f) .The core levels of Se $3d_{3/2}$ and 3d $_{5/2}$ feature a prominent doublet at 54.20 and 55.00 eV (Figure S13g) respectively which can be attributed to the Se^{2-} of $MoSe_2$. The presence of $MoSe_2$ is in agreement with the Raman observation of re-formation of $MoSe_2$ after 1.7 V in the first charge[11] (Figure S13a). Two other small doublets are presents. One is nearly negligible with Se $3d_{3/2}$ and 3d $_{5/2}$ core levels at 55.60 and 56.40 eV respectively can be attributed to the presence of elemental selenium, [20] while a second one at 52.9 eV and 53.6 eV can be attributed to metallic selenide compounds such as Na_2Se (Figure S13g). A small amount of this compound along with some Mo metal after the end of the first discharge cycle are therefore formed.[21]

We can therefore conclude that the final product at the end of the first cycle could be a mixture of partially sodiated 2H-$MoSe_2$, Mo metal, elemental Se and unreacted Na_2Se. [11] The Se $3_{d5/2}$

and Se $3d_{3/2}$ peaks at 53,0 eV and 53.63 eV are generally observed in metallic selenide compounds, and suggest that the residue of Na_2Se emerges simultaneously with the Mo metal after the end of the discharge.[21] From the discussion above, the final product at the end of the first cycle could be a mixture of partially sodiated 2H-$MoSe_2$, Mo metal, elemental Se and unreacted Na_2Se (Figure S13 and Figure S20). In addition, volume expansion was found during cycling, as shown in Figure 5c. With the intercalation of Na^+, the volume of $MoSe_2$ gradually expanded and reached a maximum at 0 V, and then shrunk during desodiation, yet cannot be fully recovered at 3.0 V. Figure h and i illustrate the variation of electrode reflectance with potential during cycling. It is evident that the changes in reflectance occur only during the sodiation (cathodic cycle) and desodiation (anodic cycle) processes. During sodiation, the intensity of reflectance gradually decreases, whereas it increases during desodiation. This can correlate with the structural changes of electrode material at different sodiation states and similar phenomena have been observed in the de(lithiation) process of oxide materials. It is also worth mentioning that the intensity of reflectance cannot fully recover at the end of the first cycle, which further confirms a large number of Na ions frozen in the electrode after the first cycle.

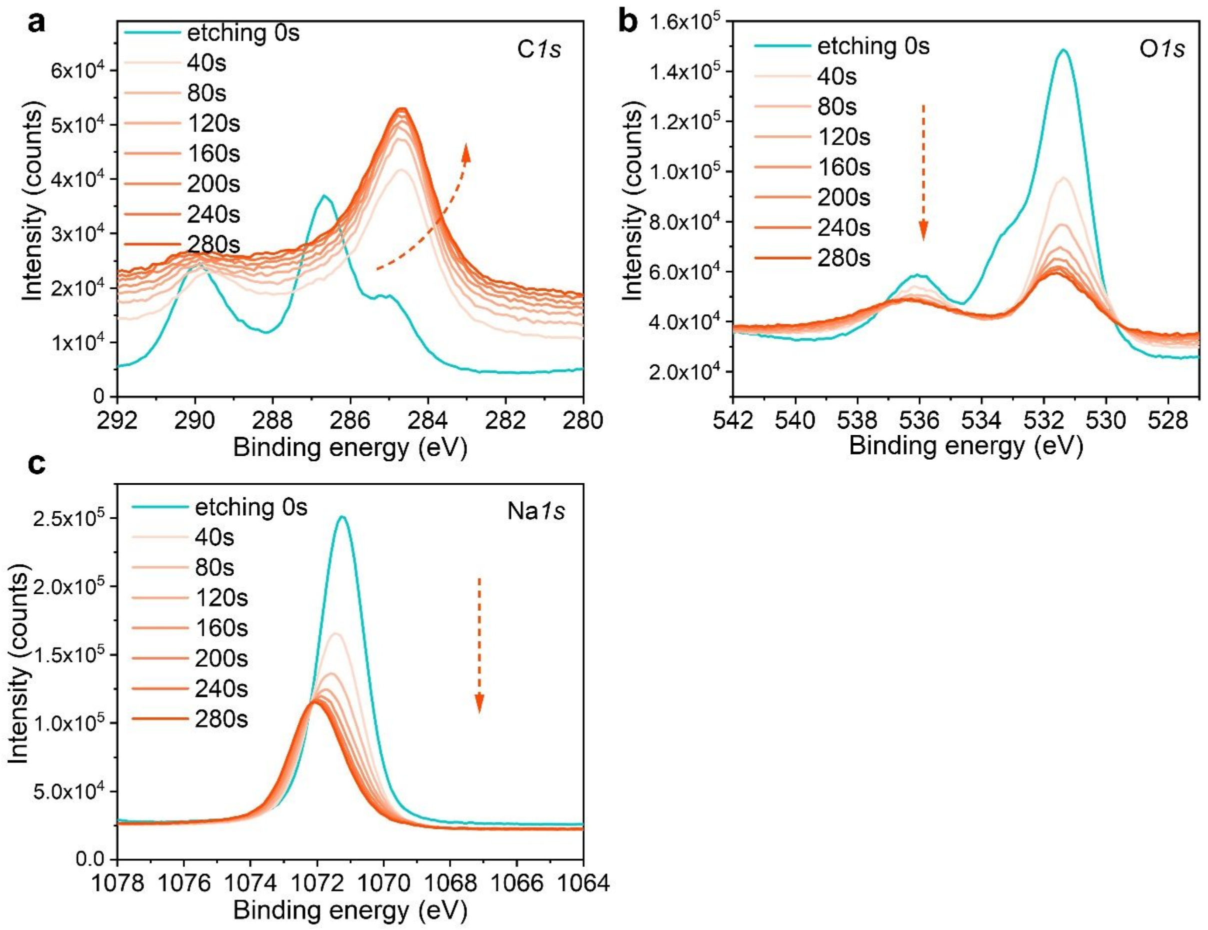

Figure S14 High resolution XPS spectrum of (a) C 1s, (b) O 1s, (c) Na 1s before(etching 0 seconds) and after Ar etching (40-280 seconds).

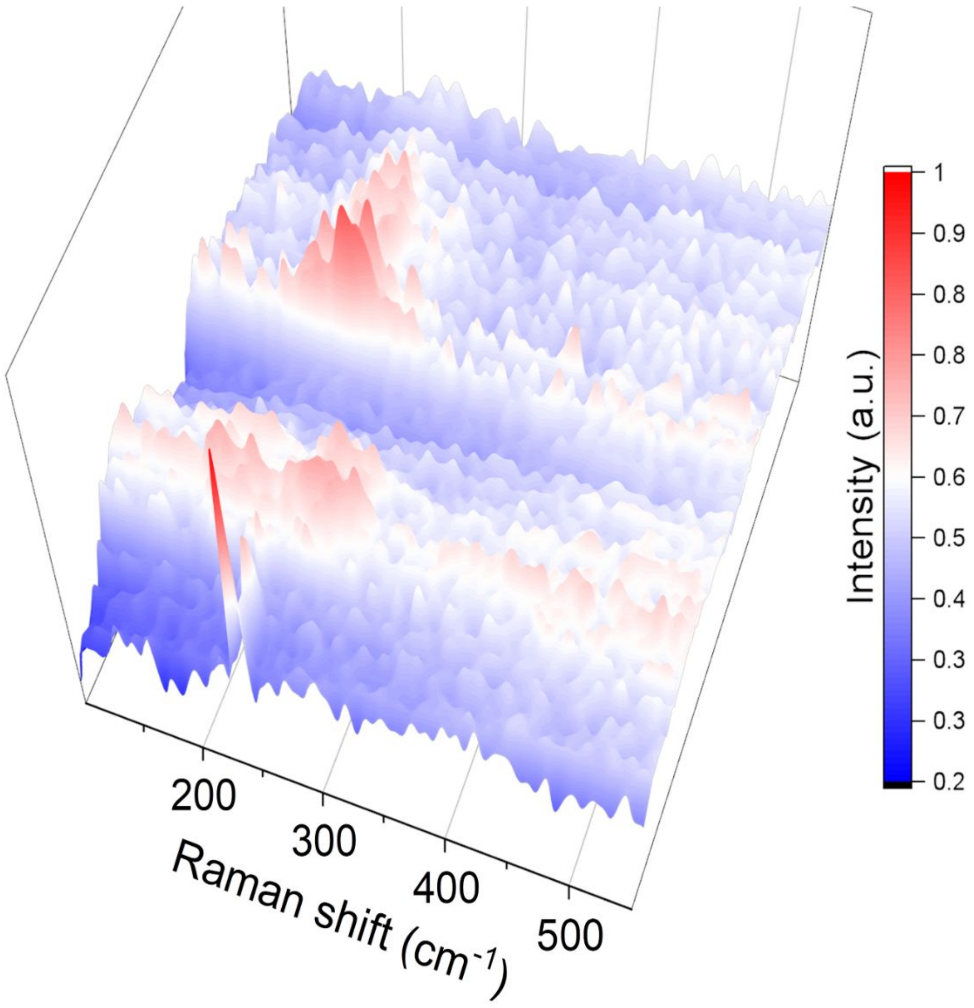

Figure S15 Corresponding 2D map of the operando Raman shown in Figure S13a.

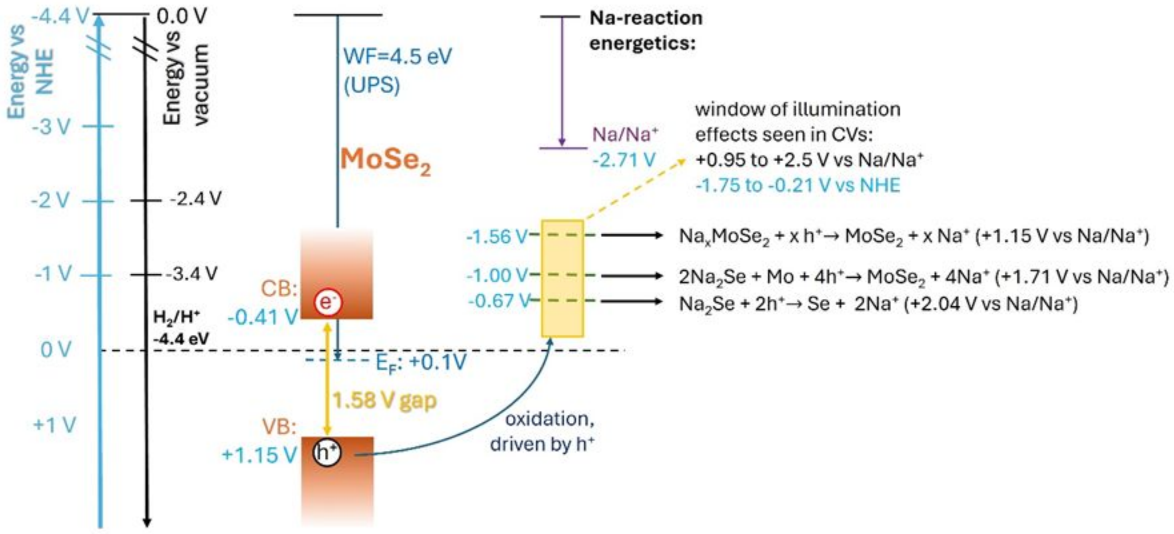

Figure S16. The energy level band diagram of $MoSe_2$ during photo-assisted charging process of Na-ion battery.

To clarify the photo-recharging mechanism of the battery, we added Fig: S16, including band positions extracted by UPS, and all other reaction mechanisms and energetics. Indeed, the photo-generated charge carriers generated in the $MoSe_2$ do not have the right band positions to recharge the Na anode. However, the holes recharge the cathode material ($MoSe_2$) by depleting it from Na, and thereby increasing the internal voltage against Na. As such, next cycle use again Na from the anode upon discharge. The battery is hence photo rechargeable as long as excess Na is provided from the anode, having typically larger capacity than the cathode.

Aside, we propose the following mechanistic figure to visualize the function of the battery upon photocharging, and on discharge (Figure S17).

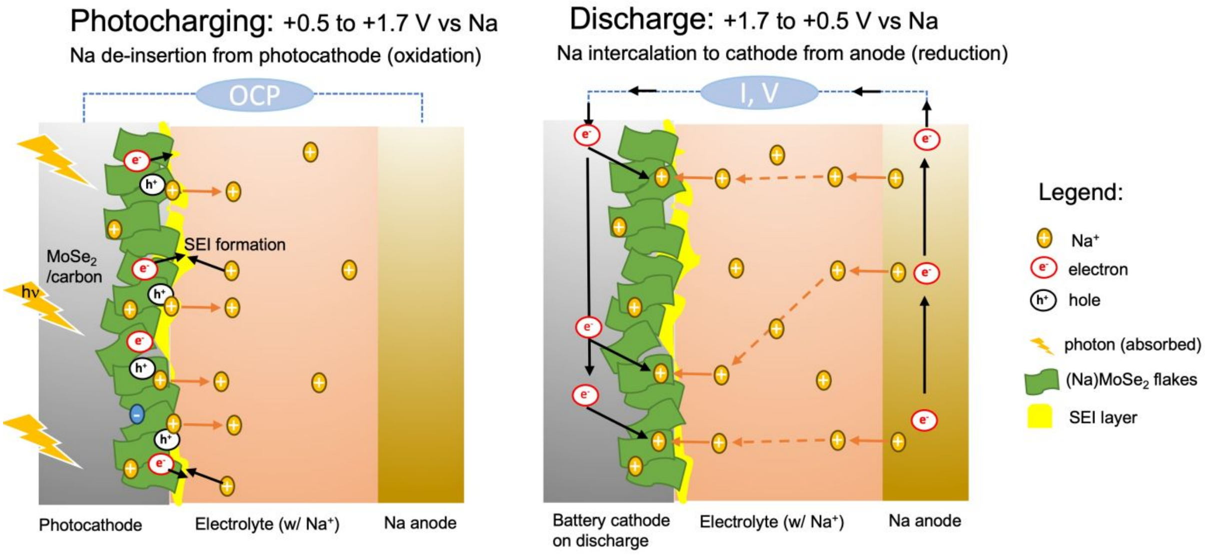

Figure S17. Proposed photo battery mechanism.

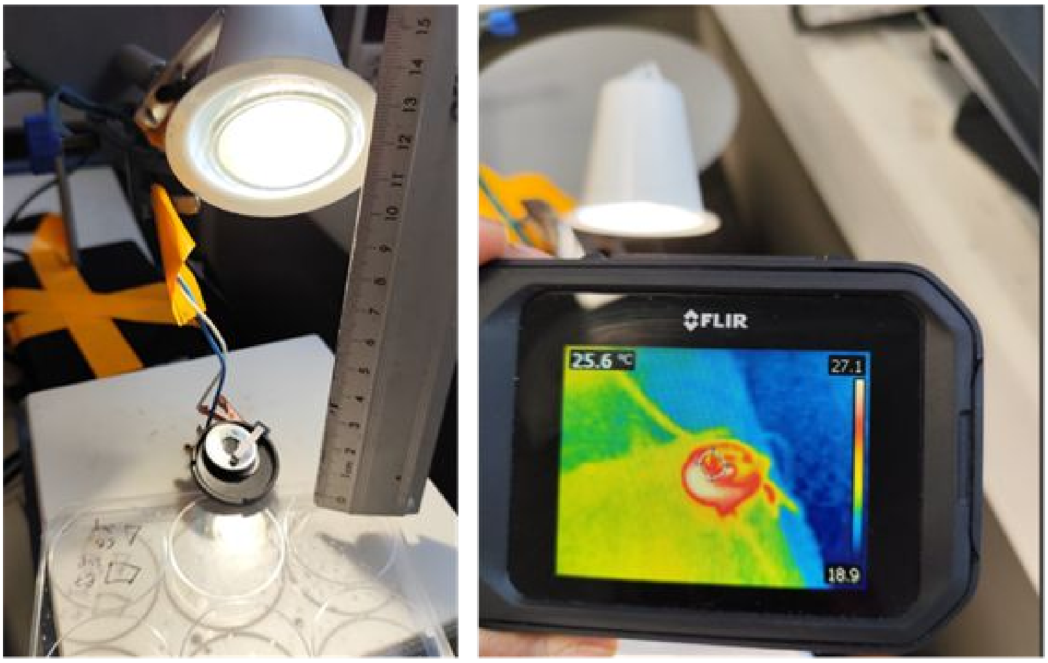

Figure S18. Light exposure on the photo-coincell and temperature measurement of the photo-cincell after light illumination for 46 h.

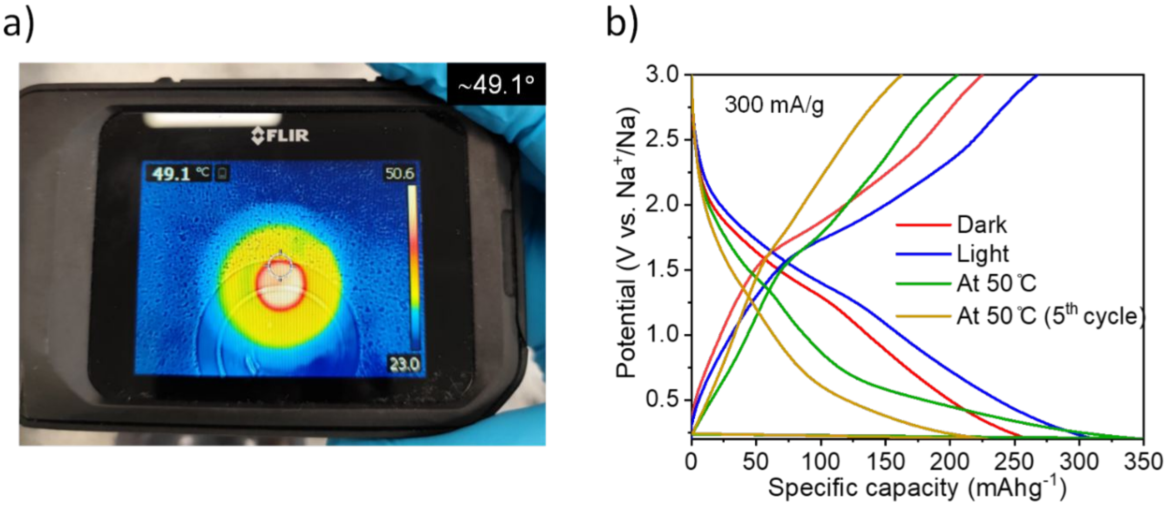

Figure S19. External heating of Na-ion battery at 50 °C and charge-discharge characterization comparing dark- and light-assisted charge-discharge performance.

To address possible heating effects, we have measured the temperature of the coin cell and the active material under illumination of household LED lamp after 46 hours of continuous illumination. We have measured a temperature of 25.6 °C inside the cell, thus excluding that heating could be the cause of the increased capacity under illumination (Figure S18). This

result is reasonable since we have used a low power conventional LED lamp (11.3 mW/cm^{2,} estimated by a photodiode measurement at 590nm, overall range: 940-450 nm) with an illumination distance of 8-12 cm to coin cell (as shown in Figure S18). Moreover, we have performed GCD experiments while heating up the coin cell at 50 °C (Figure S19). The cell displays a slight capacity increase after the first cycle (~1.11%) compared to the capacity obtained withing heating and in the dark, while a significant decrease in the following cycles. This first cycle increase is lower than the capacity enhancement observed after the first cycle in the battery under illumination. The following cycles show a significant degradation of the battery at the temperature of 50 °C. Thus, the increase in capacity of our battery under illumination can not be explained by an increase in temperature since this led to a quick degradation of the battery.

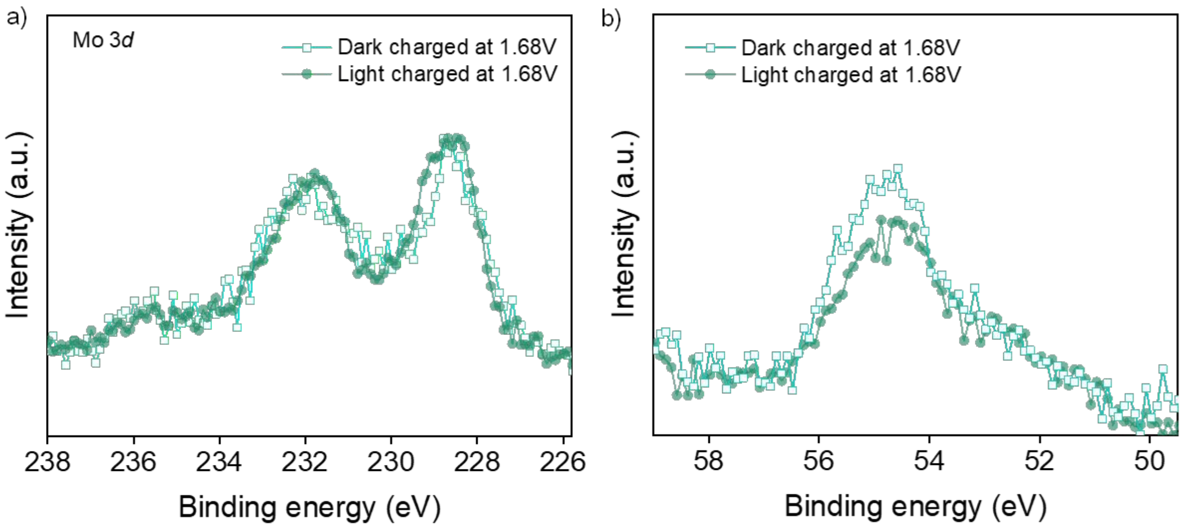

Figure S20; XPS spectra of Mo 3d$_{3/2}$ and d$_{5/2}$ and Se 2p$_{1/2}$ and 2p$_{3/2}$ from photocathodes. The dark green colour spectrum has been collected after light assisted charging (at 1.68V) after being discharged at 0.2V while the light green colour spectrum after and dark charging (at 1.68V) after being discharged at 0.2V.

The binding energy position of the core level of Mo 3d (3d5/2 and 3d3/2) provide insightful information about the crystal phase, 1T[17] versus 2H[17, 22], and the presence of possible intercalated Na ions[16, 17] . After a charging under light, 3 components are present: a

predominant one at 229.2 eV, one at 228.5 eV and one at 228.0 eV. The 229.2eV component can be attribute to the presence of the 2H phase suggesting that under light illumination the 2H phase is restored form the 1T phase and Na ion and pushed out from the structure. 228.5 eV can be attributed to a Na intercalated phase of 1T $MoSe_2$ [16]. The 228 eV component can be attributed to the 1T $MoSe_2$: still this phase is presents suggesting that not all the light can convert the 1T into 2H. Interestingly, upon charging in the dark, as expected. there is a predominant presence of Na-intercalated 1T MSe_2 (228.5 eV) and 1T $MoSe_2$ (228.0eV). These results are also in agreement with the Raman observations.

Solar-to-energy conversion efficiency

A solar to energy conversion efficiency is given and compared. This efficiency is defined as electrical energy out/light energy in [23]:

$$\eta_{overall} = \frac{E_{output}}{E_{input}} = \frac{\int V dt \times I_{CD}}{A \times P_{in} \times t}$$

We measured the incident light energy with a photodiode. Setting the wavelength to 590 nm as reasonable average value, we obtain 8.8mW [11.3 mW/cm^2, 22.6 mW on our cell with a 0.5cm^2 window]. Over 46h of illumination, this equals 934J of light energy delivered.

The integrated energy obtained by discharging the battery from 1.68V at a current of 0.036mA is 1.79J. As ratio between the two, the solar to energy conversion efficiency hence is ~0.19 %. We have also calculated the photon-to-charge conversion efficiency (photonic efficiency). Assuming again an average photon energy of 2.1 eV (590 nm), the incident energy of 934J equals $2.77*10^{21}$ photons. Discharge over 17.18h at 0.036 mA yields 2.23C, or $1.39*10^{19}$ electrons. The ratio (photonic efficiency) is ~0.5%.

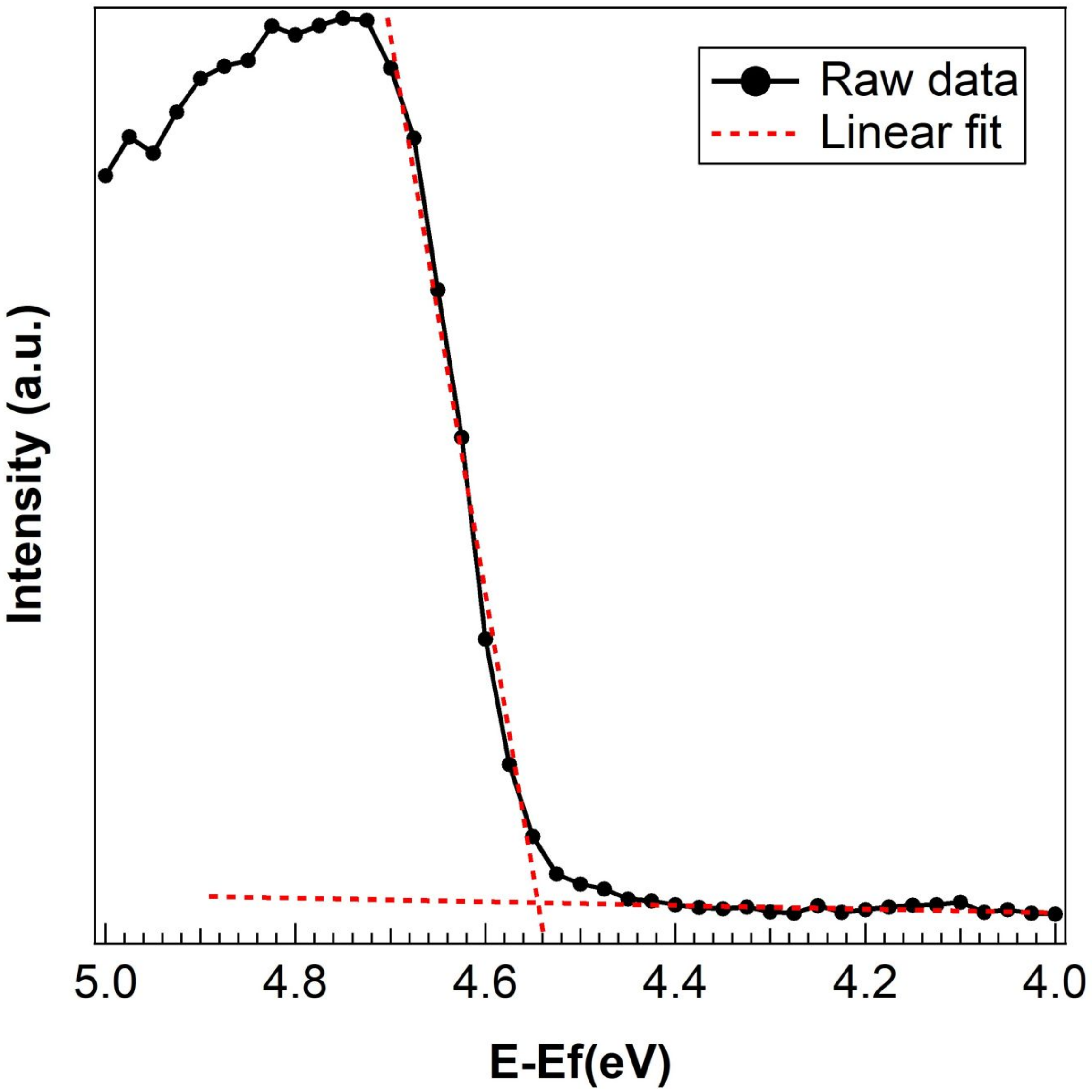

Figure S21: The work function (WF) measurement was carried out at room temperature using a He discharge lamp with a pass energy of 2 eV and an overall energy resolution of 0.15 eV, on a VG ESCALAB MKII. The energy scale was calibrated using a freshly cleaned polycrystalline Au foil. The sample's work function cut-off was determined to be 4.55 eV by the intersection of linear fits.

SI REFERENCES:

(1) Gerchman, D.; Alves, A. K. Solution-processable exfoliation and suspension of atomically thin WSe2. *J Colloid Interface Sci* **2016**, *468*, 247-252. DOI: 10.1016/j.jcis.2016.01.073 From NLM PubMed-not-MEDLINE.

(2) Ge, P.; Hou, H.; Banks, C. E.; Foster, C. W.; Li, S.; Zhang, Y.; He, J.; Zhang, C.; Ji, X. Binding MoSe2 with carbon constrained in carbonous nanosphere towards high-capacity and ultrafast Li/Na-ion storage. *Energy Storage Materials* **2018**, *12*, 310-323.

(3) Diskus, M.; Nilsen, O.; Fjellvåg, H. Growth of thin films of molybdenum oxide by atomic layer deposition. *Journal of Materials Chemistry* **2011**, *21* (3), 705-710.

(4) Yousaf, M.; Wang, Y. S.; Chen, Y. J.; Wang, Z. P.; Firdous, A.; Ali, Z.; Mahmood, N.; Zou, R. Q.; Guo, S. J.; Han, R. P. S. A 3D Trilayered CNT/MoSe2/C Heterostructure with an Expanded MoSe2 Interlayer Spacing for an Efficient Sodium Storage. *Advanced Energy Materials* **2019**, *9* (30), 1900567. DOI: ARTN 1900567

10.1002/aenm.201900567.

(5) Huang, X. L.; Zhou, C.; He, W.; Sun, S.; Chueh, Y. L.; Wang, Z. M.; Liu, H. K.; Dou, S. X. An Emerging Energy Storage System: Advanced Na-Se Batteries. *ACS Nano* **2021**, *15* (4), 5876-5903. DOI: 10.1021/acsnano.0c10078 From NLM PubMed-not-MEDLINE.

(6) Brezesinski, T.; Wang, J.; Tolbert, S. H.; Dunn, B. Ordered mesoporous α-MoO3 with iso-oriented nanocrystalline walls for thin-film pseudocapacitors. *Nature materials* **2010**, *9* (2), 146-151.

(7) Cao, R.; Zhuang, Q.-C.; Tian, L.-L.; Qiu, X.-Y.; Shi, Y.-L. Electrochemical impedance spectroscopic study of the lithium storage mechanism in commercial molybdenum disulfide. *Ionics* **2014**, *20* (4), 459-469.

(8) Sokolikova, M. S.; Mattevi, C. Direct synthesis of metastable phases of 2D transition metal dichalcogenides. *Chemical Society Reviews* **2020**, *49* (12), 3952-3980. Kwon, I. S.; Kwak, I. H.; Debela, T. T.; Abbas, H. G.; Park, Y. C.; Ahn, J.-p.; Park, J.; Kang, H. S. Se-Rich MoSe2 Nanosheets and Their Superior Electrocatalytic Performance for Hydrogen Evolution Reaction. *ACS Nano* **2020**, *14* (5), 6295-6304. DOI: 10.1021/acsnano.0c02593. Naz, M.; Hallam, T.; Berner, N. C.; McEvoy, N.; Gatensby, R.; McManus, J. B.; Akhter, Z.; Duesberg, G. S. A New 2H-2H′ /1T Cophase in Polycrystalline MoS2 and MoSe2 Thin Films. *ACS Applied Materials & Interfaces* **2016**, *8* (45), 31442-31448. DOI: 10.1021/acsami.6b10972. Gupta, U.; Naidu, B. S.; Maitra, U.; Singh, A.; Shirodkar, S. N.; Waghmare, U. V.; Rao, C. N. R. Characterization of few-layer 1T-MoSe2 and its superior performance in the visible-light induced hydrogen evolution reaction. *APL Materials* **2014**, *2* (9). DOI: 10.1063/1.4892976 (acccessed 7/13/2024).

(9) Voiry, D.; Mohite, A.; Chhowalla, M. Phase engineering of transition metal dichalcogenides. *Chemical Society Reviews* **2015**, *44* (9), 2702-2712.

(10) Mahrouche, F.; Rezouali, K.; Mahtout, S.; Zaabar, F.; Molina-Sánchez, A. Phonons in WSe2/MoSe2 van der Waals heterobilayers. *physica status solidi (b)* **2022**, *259* (1), 2100321. Jiang, M.; Hu, Y.; Mao, B.; Wang, Y.; Yang, Z.; Meng, T.; Wang, X.; Cao, M. Strain-regulated Gibbs free energy enables reversible redox chemistry of chalcogenides for sodium ion batteries. *Nat. Commun.* **2022**, *13* (1), 1-13.

(11) Plewa, A.; Kulka, A.; Hanc, E.; Sun, J.; Nowak, M.; Redel, K.; Lu, L.; Molenda, J. Abnormal Phenomena of Multi - Way Sodium Storage in Selenide Electrode. *Advanced Functional Materials* **2021**, *31* (29). DOI: 10.1002/adfm.202102406.

(12) Herwig, C.; Schnell, M.; Becker, J. Raman band of matrix isolated NaMSeN clusters. *Chemical physics letters* **2004**, *385* (5-6), 462-466.

(13) Xu, Y.; Liu, X.; Su, H.; Jiang, S.; Zhang, J.; Li, D. Hierarchical bimetallic selenides CoSe2 - MoSe2/rGO for sodium/potassium - ion batteries anode: insights into the intercalation and conversion mechanism. *Energy & Environmental Materials* **2022**, *5* (2), 627-636.
(14) Goldbach, A.; Johnson, J.; Meisel, D.; Curtiss, L.; Saboungi, M.-L. On the constituents of aqueous polyselenide electrolytes: a combined theoretical and Raman spectroscopic study. *Journal of the American Chemical Society* **1999**, *121* (18), 4461-4467.
(15) Sokolikova, M. S.; Cheng, G.; Och, M.; Palczynski, P.; El Hajraoui, K.; Ramasse, Q. M.; Mattevi, C. Tuning the 1T′ /2H phases in WxMo1−xSe2 nanosheets. *Nanoscale* **2023**, *15* (6), 2714-2725, 10.1039/D2NR05631C. DOI: 10.1039/D2NR05631C.
(16) Kondekar, N. P.; Boebinger, M. G.; Woods, E. V.; McDowell, M. T. In Situ XPS Investigation of Transformations at Crystallographically Oriented MoS2 Interfaces. *ACS Applied Materials & Interfaces* **2017**, *9* (37), 32394-32404. DOI: 10.1021/acsami.7b10230.
(17) Papageorgopoulos, C. A.; Jaegermann, W. Li intercalation across and along the van der Waals surfaces of MoS2(0001). *Surface Science* **1995**, *338* (1), 83-93. DOI: https://doi.org/10.1016/0039-6028(95)00544-7.
(18) Sokolikova, M. S.; Cheng, G.; Och, M.; Palczynski, P.; El Hajraoui, K.; Ramasse, Q. M.; Mattevi, C. Tuning the 1T′ /2H phases in W x Mo 1− x Se 2 nanosheets. *Nanoscale* **2023**, *15* (6), 2714-2725.
(19) Ithurria, S.; Dubertret, B. Quasi 2D colloidal CdSe platelets with thicknesses controlled at the atomic level. *Journal of the American Chemical Society* **2008**, *130* (49), 16504-16505.
(20) Niu, F. E.; Yang, J.; Wang, N. N.; Zhang, D. P.; Fan, W. L.; Yang, J.; Qian, Y. T. MoSe2-Covered N,P-Doped Carbon Nanosheets as a Long-Life and High-Rate Anode Material for Sodium-Ion Batteries. *Advanced Functional Materials* **2017**, *27* (23). DOI: ARTN 1700522

10.1002/adfm.201700522.
(21) Naveau, A.; Monteil-Rivera, F.; Guillon, E.; Dumonceau, J. Interactions of aqueous selenium (− II) and (IV) with metallic sulfide surfaces. *Environmental science & technology* **2007**, *41* (15), 5376-5382. Riha, S. C.; Johnson, D. C.; Prieto, A. L. Cu2Se nanoparticles with tunable electronic properties due to a controlled solid-state phase transition driven by copper oxidation and cationic conduction. *Journal of the American Chemical Society* **2011**, *133* (5), 1383-1390.
(22) Eda, G.; Yamaguchi, H.; Voiry, D.; Fujita, T.; Chen, M.; Chhowalla, M. Photoluminescence from Chemically Exfoliated MoS2. *Nano Letters* **2011**, *11* (12), 5111-5116. DOI: 10.1021/nl201874w.
(23) Dong, Q.; Wei, M.; Zhang, Q.; Xiao, L.; Cai, X.; Zhang, S.; Gao, Q.; Fang, Y.; Peng, F.; Yang, S. Photoassisted Li-ion de-intercalation and Niδ+ valence conversion win-win boost energy storage performance in Ni/CdS@Ni3S2-based Li-ion battery. *Chemical Engineering Journal* **2023**, *459*, 141542. DOI: https://doi.org/10.1016/j.cej.2023.141542.
